# Evaluating the mechanical behavior of plastic waste modified asphalt using optimized machine learning approaches

**DOI:** 10.1038/s41598-025-24107-3

**Published:** 2025-11-10

**Authors:** Tariq Alqubaysi, Muhammad Zeeshan Qureshi, Inamullah Inam, Tariq Ali, Khaled Mohamed Elhadi, Ahmed A. Alawi Al-Naghi, Hawreen Ahmed

**Affiliations:** 1 Department of Civil Engineering, College of Engineering, Northern Border University, Arar, 73222 Saudi Arabia; 2 Department of Civil Engineering, University of Engineering and Technology, Taxila, Pakistan; 3 Department of Civil Engineering, Engineering Faculty, Laghman University, Mehtarlam, Afghanistan; 4 Department of Civil Engineering, Swedish College of Engineering and Technology, Wah, 47080 Pakistan; 5 Civil Engineering Department, College of Engineering, King Khalid University, Abha, Saudi Arabia; 6 Center for Engineering and Technology Innovations, King Khalid University, Abha, 61421 Saudi Arabia; 7 Civil Engineering Department, University of Ha’il, Ha’il, 55476 Saudi Arabia; 8 Department of Highway and Bridge Engineering, Technical Engineering College, Erbil Polytechnic University, Erbil, 44001 Iraq

**Keywords:** Plastic modified asphalt, Marshall stability, Marshall flow, Machine learning, Model interpretability, Engineering, Civil engineering

## Abstract

The growing environmental challenges associated with plastic waste disposal and the need for sustainable pavement construction practices have prompted significant research interest in incorporating recycled plastics into asphalt mixtures. However, accurately predicting the performance characteristics of plastic-modified asphalt mixtures, particularly Marshall Stability (MS) and Marshall Flow (MF), remains a critical yet challenging task due to complex nonlinear relationships between mixture constituents. This study addresses this issue by developing reliable predictive models using machine learning techniques including Support Vector Machine (SVM), Decision Tree (DT), Random Forest (RF), Extreme Gradient Boosting (XGB), and Light Gradient Boosting Machine (LGBM), further optimized through Particle Swarm Optimization (PSO). A comprehensive dataset comprising 210 samples of plastic-modified asphalt mixtures was utilized, incorporating inputs such as plastic content and size, bitumen content, maximum aggregate size, mixing temperature, and compaction effort (number of blows), to predict MS and MF as outputs. Results showed that the PSO-optimized XGB model achieved the highest accuracy, yielding R^2^ values of 0.82 for MS and 0.83 for MF. Model interpretability was enhanced using advanced techniques such as SHapley Additive exPlanations (SHAP), Partial Dependence Plots (PDP), Individual Conditional Expectation (ICE) plots, and Taylor diagrams, quantitatively highlighting optimal plastic particle sizes (2.5–4 mm), bitumen content (5.3–5.5%) and plastic content (20–30%). These findings provide actionable insights that support safer and longer-lasting pavements, promote the sustainable reuse of waste plastics, and enable cost-effective mix design strategies for modern asphalt construction.

## Introduction

Asphalt pavements form the backbone of roadway infrastructure worldwide, yet they are continually subjected to deterioration from heavy traffic loads and environmental stresses. In fact, asphalt concrete comprises roughly 85% of paved roads globally^1–3^, and common distress modes such as rutting at high temperatures and cracking at low temperatures often emerge over a pavement’s service life^4^. Frequent maintenance and rehabilitation are required to address these issues, indicating a pressing need for more durable asphalt mixtures. At the same time, the world faces an escalating problem of plastic waste accumulation. Plastics are highly resistant to degradation and can persist for decades as pollutants^5,6^; globally, plastic waste has roughly doubled since 2000 to ~ 353 Mt in 2019, yet only about 9% is recycled, with much of the remainder mismanaged in landfills or the natural environment^7^. Empirical studies likewise report measurable performance gains from recycled-plastic modifiers e.g., ~ 32% higher Marshall stability at 7.5% PET and ~ 48% higher stability at 6% LDPE alongside systematic evidence of improved rutting and fatigue resistance relative to control mixes^8–10^. These coupled infrastructure and waste-management pressures have motivated the development of plastic-modified asphalt as a synergistic solution.

Bitumen, the viscoelastic binder in asphalt concrete, though typically constituting only ~ 5% of the mixture by weight, plays a critical role in pavement performance. Its properties largely govern the mixture’s response to loading and temperature. Polymer modification of bitumen has become a well-established strategy to enhance asphalt performance and extend pavement longevity. By incorporating polymers into bitumen, the binder’s elasticity and stiffness can be improved, helping the pavement resist rutting, cracking, and aging^4,11^. Traditionally, engineered polymers such as styrene–butadiene–styrene (SBS) has been added to bitumen to improve high-temperature stability and low-temperature flexibility^11,12^. In recent years, attention has turned to polymer modifiers derived from waste materials, especially waste plastics, as a dual-benefit innovation. Using recycled plastics such as PET and LDPE as asphalt modifiers provides a productive outlet for plastic waste while empirically improving asphalt performance. Laboratory studies report significant gains in mechanical properties, for example, Marshall stability increased by ~ 48% at ~ 6% LDPE and by ~ 31–32% at ~ 7.5% PET, with systematic reviews also confirming improvements in rutting resistance, fatigue life, moisture tolerance, and high-temperature stability compared with conventional mixtures^4,9,13–17^. For instance, adding waste polyethylene has been shown to increase mixture stiffness and stability at elevated service temperatures, thereby mitigating permanent deformation under load^9^. These improvements are attributed to plastic acting as a polymeric reinforcement that enhances binder cohesion, thermal stability, and elasticity^18^. At the same time, the literature notes important deployment challenges such as dispersion and binder-plastic compatibility, storage stability, and possible low-temperature brittleness that can offset benefits if left unmanaged^15^. Consequently, recycled plastics should be regarded as conditionally beneficial modifiers, whose performance advantages are realized when challenges such as dispersion, compatibility, and low-temperature brittleness are appropriately managed. When applied under such conditions, plastic-modified asphalt not only enhances durability and rutting resistance but also contributes to circular economy goals by diverting substantial volumes of plastic from landfills, thereby addressing both infrastructure performance and environmental sustainability^13,15,19,20^.

Beyond plastics, numerous other additives have been explored to improve asphalt mixture performance. Crumb rubber derived from waste tires is a well-known modifier that can improve asphalt’s high-temperature, low-temperature, and fatigue performance. Rubber-modified asphalts have been widely used in practice, increasing the elastic properties of ground tire rubber to increase rutting resistance and reduce road noise^21–23^. Fibers (such as cellulose fibers, glass fibers, or polymer fibers) have also been used to reinforce asphalt mixtures as these can inhibit crack propagation and reduce asphalt binder drain-down, thereby enhancing mixture stability and indirect tensile strength^24^. Likewise, nanomaterials (e.g., nano-silica, nano-clays, nano-calcium carbonate) have shown promise in further improving asphalt performance. The addition of nano-sized additives can refine the binder’s microstructure and has been found to increase the high-temperature stability and rutting resistance of asphalt binders, as well as improve aging resistance and moisture susceptibility in some cases^25^. Each of these modifiers from rubbers to fibers to nanoparticles targets specific deficiencies in conventional asphalt. Nonetheless, among the variety of modifiers, recycled plastic waste has gained special interest due to its potential to tackle environmental waste while offering performance benefits comparable to commercial polymers^15^. This has led to a proliferation of research on plastic-reinforced or plastic-modified asphalt mixtures in the past few years.

In parallel with material innovations, the field of asphalt technology has seen a growing adoption of data-driven approaches. Machine learning (ML) techniques are increasingly employed to predict asphalt mixture properties and optimize mix designs. Traditional trial-and-error laboratory methods for evaluating properties like Marshall Stability and Flow can be time-consuming and resource-intensive. Researchers are thus turning to ML models that learn from experimental data to forecast these properties more efficiently^26^. A range of supervised ML algorithms have been applied in this context, including decision tree learners, support vector machines (SVMs), artificial neural networks (ANNs), and ensemble methods such as random forests and gradient boosting algorithms. For instance, Gul et al. utilized a Supervised Machine Learning Algorithms (ANN, ANFIS and MEP) to predict the Marshall characteristics such as Marshall stability and Flow of asphalt mixes utilizing 343 datapoints, achieving excellent accuracy (R² ≈ 0.80) for both stability and flow prediction by MEP^27^. Similarly, other studies have used ML to model the outcomes of Marshall mix design, to estimate optimal bitumen content, and even to predict performance metrics like stiffness or indirect tensile strength based on mixture composition^28–30^. In the specific domain of plastic-modified asphalts, initial studies have also emerged. For example, some researchers have modeled the Marshall Stability of plastic-reinforced asphalt using algorithms like neural networks and regression trees, highlighting the feasibility of ML in this niche^31^. In one investigation, researchers developed a range of soft computing models, Random Forest (RF), Random Tree (RT), Bagging RT, Bagging RF, and Artificial Neural Networks (ANN) using 90 experimental observations to predict the Marshall Stability of plastic-modified asphalt concrete^32^. Their models incorporated inputs such as bitumen content, plastic content, bitumen grade, and plastic size. Among the tested algorithms, the RF model achieved superior prediction performance, with a correlation coefficient (R) of 0.942 for training and 0.896 for testing, alongside low RMSE and MAE values. Notably, sensitivity analysis identified plastic size as a critical predictor, emphasizing the importance of polymer granulation in asphalt mix design. Similarly, another study utilized 265 data points to train ensemble learning models, including Gradient Boosting, XGB, and Bagging Regressor, for predicting Marshall Stability of plastic-reinforced asphalt^33^. XGB demonstrated the strongest prediction capability with R values of 0.95 (training) and 0.84 (testing). This study also applied SHapley Additive exPlanations (SHAP) to interpret model predictions, confirming the dominant influence of bitumen content and plastic particle size on the resulting asphalt stability.

Despite the success of ML models in predicting asphalt properties, challenges remain in maximizing their accuracy and interpretability. Tuning complex ML models for peak performance often requires optimizing hyperparameters (e.g. tree depths, learning rates, kernel parameters), which can significantly influence prediction accuracy. To address this, researchers have started integrating optimization algorithms with ML. Metaheuristic optimization techniques such as Genetic Algorithms (GA), Particle Swarm Optimization (PSO), and others have been used to automatically search for optimal model parameters. In pavement engineering applications, for instance, PSO has been combined with support vector regression to improve pavement performance prediction models^34^. In another study, a hybrid Extreme Gradient Boosting model optimized via Whale Optimization Algorithm (WOA) outperformed standard models in predicting asphalt mixture dynamic modulus^35^. These prior studies demonstrate that coupling ML algorithms with metaheuristic optimization significantly enhances both predictive accuracy and generalization. For example, Li et al.^34^ showed that PSO-optimized SVR outperformed standard SVR in predicting asphalt rutting performance on expressways, achieving higher reliability and reduced error. Similarly, Zhang et al.^35^ reported that WOA-XGBoost achieved an R^2^ above 0.99 for dynamic modulus prediction while also identifying key mixture factors using SHAP and PDP. Together, these findings substantiate that hybrid ML-optimization approaches are not only more accurate but also provide actionable insights for pavement mixture design. However, such approaches are still relatively uncommon in asphalt materials research. The use of PSO to fine-tune models like XGB for asphalt mixture property prediction has seen limited exploration in literature, especially for modified asphalt systems. Another critical aspect gaining attention is the explainability of machine learning models. Complex models (e.g. ensemble boosted trees or deep networks) often act as “black boxes,” making it difficult for engineers to understand why a certain prediction is made. This lack of transparency can hinder trust and acceptance of ML solutions in engineering practice^36^. To overcome this, explainable artificial intelligence (XAI) techniques are being adopted. Two powerful XAI tools are SHAP and Partial Dependence Plots (PDP)^36^.

While prior research has examined plastic-modified asphalt mixtures and the application of machine learning to predict asphalt properties, there remains a notable gap in integrating these advancements. Few studies have combined state-of-the-art ML modeling with optimization algorithms and XAI techniques specifically for plastic-reinforced asphalt mixtures. Most existing investigations rely heavily on experimental characterization, and those incorporating machine learning often omit advanced hyperparameter optimization or detailed model interpretability. This gap restricts the full exploitation of modern computational tools for designing sustainable, high-performance asphalt mixtures incorporating waste plastics. To address this limitation, this research develops machine learning models to predict the Marshall Stability (MS) and Marshall Flow (MF) characteristics of plastic-reinforced asphalt mixtures, which are critical indicators of pavement strength and flexibility influencing road safety and service life. By accurately predicting these properties, the study facilitates the design of durable, cost-effective, and sustainable pavements that incorporate recycled plastics. Various algorithms including DT, RF, SVM, LGBM, and XGB are applied, with an XGB model further optimized using PSO to achieve enhanced predictive accuracy. Additionally, SHAP and PDP analyses are applied to interpret the influence of mixture parameters on Marshall performance outcomes. By integrating machine learning, optimization, and interpretability, this research introduces a comprehensive framework for effectively modeling and understanding the behavior of plastic-modified asphalt mixtures. The approach not only contributes to the growing body of knowledge on sustainable pavement technologies but also delivers practical insights for engineering applications aimed at reusing plastic waste without compromising performance standards. The findings are expected to bridge the identified research gap and facilitate the adoption of intelligent, transparent modeling practices in asphalt mix design.

## Methodology

### Data collection

This study is based on a comprehensive dataset of 210 points developed through manual extraction of experimental data from 23 peer-reviewed journal articles^37–58^ focused on plastic-reinforced asphalt mixtures^49^. The selected literature includes a wide range of studies that investigated the influence of plastic waste types, bitumen contents, and other key mix design parameters on the Marshall properties of asphalt concrete. These studies provided data either in tabular form or through graphical representations, which were digitized using appropriate tools to extract precise numerical values.

The dataset encompasses a variety of input parameters, including plastic content and plastic particle size. It also includes key mix design variables such as bitumen content, maximum aggregate size, mixing temperature, compaction effort (number of blows). These parameters are known to significantly influence the mechanical performance of asphalt mixes. The output variables of interest are Marshall Stability (measured in kilonewtons) and Marshall Flow (measured in millimeters), both of which serve as standard indicators of the strength and deformation characteristics of asphalt concrete.

### Descriptive statistical analysis and visualizations

To understand the underlying structure and variability of the compiled dataset, a comprehensive descriptive statistical analysis was conducted. This included computing measures of central tendency (mean, median, and mode), dispersion (standard deviation and variance), and distribution shape (skewness and kurtosis) for each numeric variable. A summary of these statistical descriptors is presented in Table 1, which reveals that some parameters exhibit notable skewness and kurtosis, indicating non-normal distributions that may influence the performance of machine learning algorithms.

**Table 1:** Descriptive statistics of input and output parameters.

Parameters	Min	Max	Mean	50%	Mode	Std	Variance	Skewness	Kurtosis
Plastic size (mm)	0	19	3.90	4	5	3.267	10.67	0.98	1.45
Plastic content (%)	0	29.7	7.41	6	0	6.584	43.35	1.211	1.836
Bitumen content (%)	4	7	5.18	5.2	4.5	0.699	0.489	0.489	− 0.306
Max aggregate size (mm)	9.5	25	17.51	19	19	3.384	11.45	− 0.339	− 0.149
Mixing temperature	140	185	161.5	160	160	9.861	97.23	− 0.408	0.211
Number of blows	50	75	65.95	75	75	12.042	145.02	− 0.579	− 1.681
Marshall stability (KN)	7.15	41.3	13.39	11.71	9.72	4.913	24.13	2.123	7.022
Flow (mm)	0.75	7.9	3.294	3.161	4	0.984	0.968	1.733	6.873

**Fig. 1:**
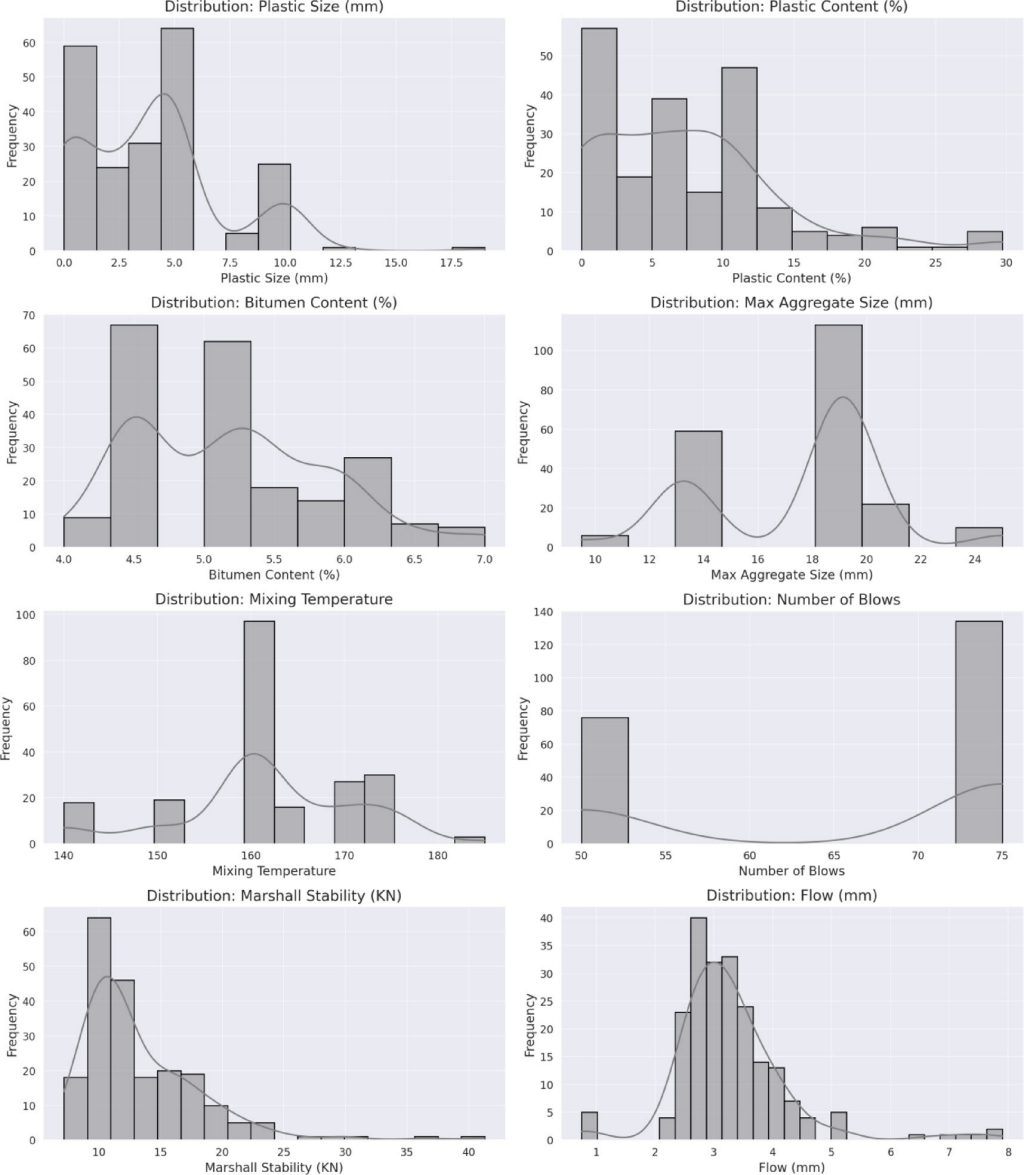
Statistical distribution histograms of input and output parameters.

To further investigate the distribution and detect potential anomalies, histograms with kernel density estimation (KDE) were generated for all numerical variables. These plots, shown in Fig. 1, illustrate the frequency distribution of each feature and highlight patterns such as skewed distributions or clustering within certain value ranges. For instance, the histogram of plastic content demonstrates a moderate right-skew, suggesting that higher plastic percentages were less frequently reported across the experimental studies. Similarly, the distribution of Marshall Stability is concentrated around mid-range values, with few extreme points at higher strengths.

### Multicollinearity analysis

Multicollinearity refers to the presence of high linear correlation between two or more independent variables in a dataset, which can adversely impact the interpretability and stability of predictive models, especially those that rely on regression-based or tree-splitting mechanisms^59^. In this study, a Pearson correlation heatmap was employed to assess the degree of linear interdependence among the input features used in the machine learning models. The correlation coefficient (r) ranges from − 1 to + 1, where values close to − 1 or + 1 indicate strong negative or positive linear relationships, respectively. For multicollinearity assessment, a common threshold is that any correlation value within the range of |r| < 0.8 is generally considered acceptable, suggesting that multicollinearity is not severe enough to require corrective measures such as dimensionality reduction or feature elimination^60^. Values beyond this threshold may indicate potential redundancy between features, which could bias model training and inflate variance.

**Fig. 2:**
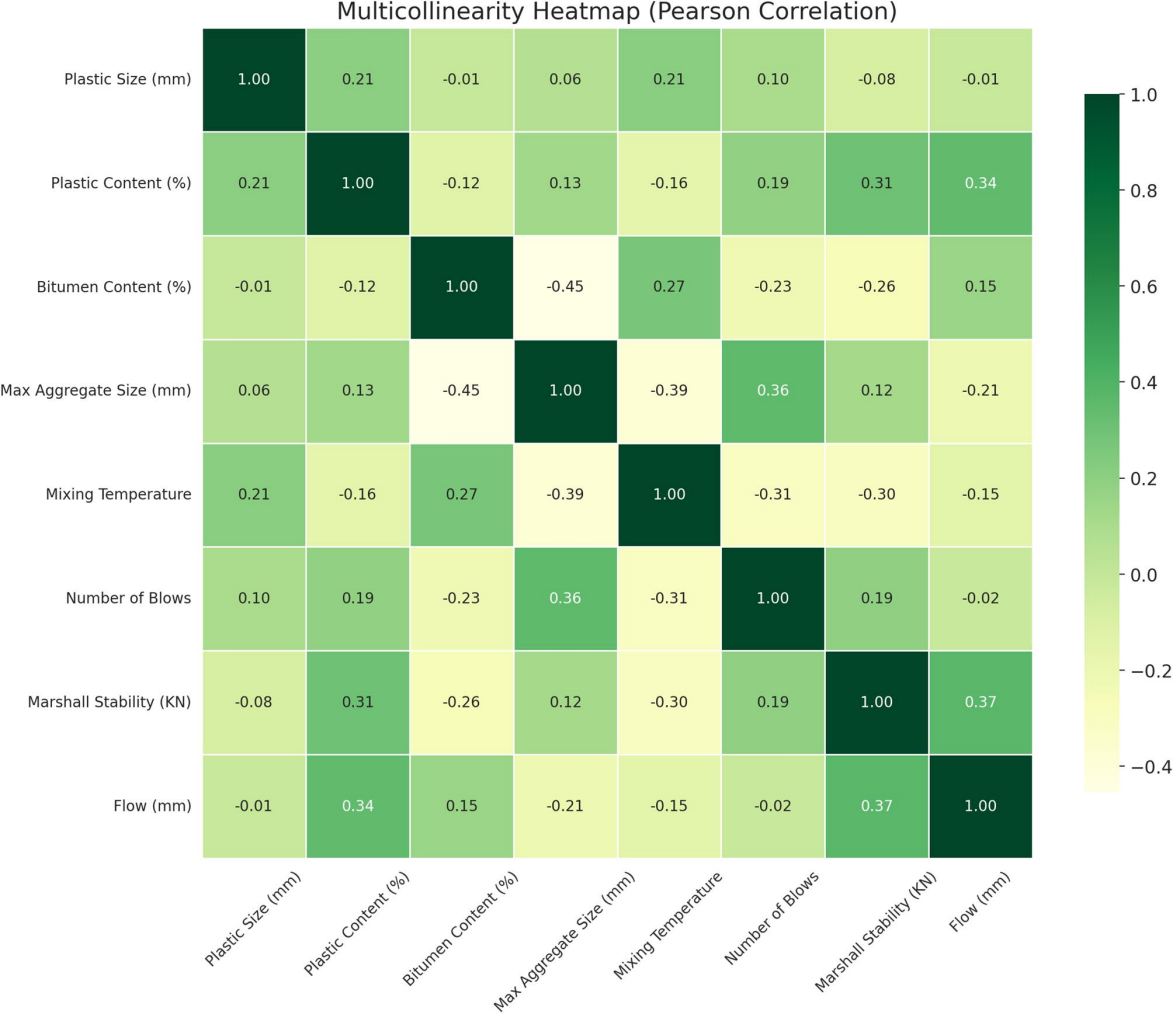
Multicollinearity heatmap.

In this study, the Pearson correlation matrix was computed for all numeric input variables and is visualized as a heatmap in Fig. 2, using a green-yellow color theme for clarity. Notable observations include a moderate positive correlation (*r* = 0.37) between Marshall Stability and Flow, as well as between Plastic Content and Flow (*r* = 0.34), suggesting that plastic dosage influences both mechanical resistance and deformation behavior. A modest negative correlation is observed between Bitumen Content and Max Aggregate Size (*r* = − 0.45), reflecting potential trade-offs in mix design configurations. Other variables, such as Plastic Size, Mixing Temperature, and Number of Blows, show minimal correlation with each other or the output variables, supporting their independent influence on mixture behavior. Overall, the multicollinearity analysis confirms that the dataset is structurally sound and free from problematic linear dependencies, providing a reliable foundation for developing machine learning models.

### Data preprocessing

Prior to model development, the dataset underwent several preprocessing steps to ensure consistency, accuracy, and readiness for machine learning implementation^61,62^. To ensure data uniformity across studies, all entries were carefully processed and standardized. Unit conversions were performed where necessary; for instance, stability values reported in kilograms were converted to kilonewtons, and flow values given in 0.01 cm were converted into millimeters. Wherever required, missing parameters were supplemented based on contextual interpretation or excluded if not applicable.

The final dataset, comprising 210 samples, was randomly divided into a training set (70%) and a testing set (30%) to evaluate model generalization performance. All machine learning models were implemented using Python in a Google Colab environment, utilizing standard libraries such as Scikit-learn, XGB, and LGBM for model training and evaluation. Data formatting, preprocessing, and visualization tasks were supported using Pandas, NumPy, Seaborn, and Matplotlib.

### Machine learning modeling

#### Decision tree

DT is a non-parametric, rule-based algorithm that recursively splits the dataset into branches based on threshold values of input features, leading to a decision outcome. It is known for its simplicity, interpretability, and ability to capture non-linear dependencies in small to medium-sized datasets^63^. However, DTs are susceptible to overfitting if not properly pruned. In pavement materials research, DTs have been used to identify influential features and develop predictive models for mixture strength and stiffness^64^.

#### Random forest

RF is an ensemble method that builds multiple decision trees on bootstrapped samples of the dataset and averages their predictions^65^. This technique improves generalization, reduces variance, and mitigates overfitting, making it a preferred choice in asphalt performance modelling. RF models are capable of handling high-dimensional data and are robust to outliers and noise^66^.

#### Support vector machine

SVM constructs an optimal hyperplane in a high-dimensional feature space to separate data points based on their labels. For regression tasks, it uses ε-insensitive loss functions and support vectors. SVM is effective in capturing complex, non-linear relationships through kernel functions and performs well on small-to-medium datasets. However, its performance is sensitive to kernel and parameter selection^27,67^.

#### Light gradient boosting machine

LGBM is a highly efficient gradient boosting framework that grows decision tree leaf-wise (instead of level-wise), enabling faster training and better accuracy in large datasets. It handles categorical features and missing values internally, reducing the need for intensive preprocessing. LGBM has shown strong performance in pavement-related predictions where training speed and scalability are crucial^68^.

#### Extreme gradient boosting

XGB is a popular and powerful ensemble method based on gradient boosting decision trees. It optimizes model performance through regularization, shrinkage, and parallel computation. Its robustness and high accuracy have made it a go-to model in civil engineering applications, particularly in predicting mechanical properties of construction materials^31,33^.

#### PSO-optimized XGB

To further improve XGB’s predictive accuracy, PSO, a bio-inspired global optimization algorithm was used to optimize its hyperparameters such as learning rate, max depth, and number of estimators^69^. PSO mimics the social behavior of birds or fish to iteratively search the solution space for the best parameter configuration. This integration has shown to significantly enhance the model’s generalization capability and reduce bias–variance trade-off^70^.In this study, the PSO itself was configured with 10 particles and a maximum of 20 iterations. The cognitive and social acceleration coefficients were set to c1 = 0.5 and c2 = 0.3, respectively, with an inertia weight of 0.9. These settings enabled PSO to efficiently search the hyperparameter space while minimizing the risk of local optima. The optimal XGB hyperparameters identified by PSO are summarized in Table 2.

### Evaluation metrics

To assess the performance of the machine learning models in predicting Marshall Stability and Flow, five commonly used evaluation metrics were employed. These metrics provide insights into the accuracy, consistency, and overall predictive quality of the models.

#### Coefficient of determination (R²)

R² quantifies how well the predicted values approximate the actual data. It ranges from 0 to 1, with higher values indicating better model fit.

$$\:{R}^{2}=\:1-\frac{{{\Sigma\:}}_{i=1}^{N}{\left({x}_{i}-{\widehat{x}}_{i}\right)}^{2}}{{{\Sigma\:}}_{i=1}^{N}{\left({x}_{i}-\stackrel{-}{x}\right)}^{2}}$$

#### Mean squared error (MSE)

MSE measures the average of the squared differences between actual and predicted values. It penalizes larger errors more than smaller ones.

$$\:MSE=\:\frac{1}{N}{\sum\:}_{i=1}^{N}{\left({x}_{i}-{\widehat{x}}_{i}\right)}^{2}$$

#### Root mean squared error (RMSE)

RMSE is the square root of MSE, providing error in the same units as the target variable. It gives a sense of how concentrated the data is around the line of best fit.

$$\:RMSE=\:\sqrt{\frac{1}{N}{\sum\:}_{i=1}^{N}{\left({x}_{i}-{\widehat{x}}_{i}\right)}^{2}}$$

#### Mean absolute error (MAE)

MAE is the average of the absolute errors between predictions and actual values. It provides a direct interpretation of prediction accuracy.

$$\:MAE=\:\frac{1}{N}{\sum\:}_{i=1}^{N}\left|{x}_{i}-{\widehat{x}}_{i}\right|$$

#### Mean absolute percentage error (MAPE)

MAPE expresses prediction error as a percentage, offering scale-independent evaluation across different magnitudes.

$$\:MAPE=\:\frac{100\%}{N}{\sum\:}_{i=1}^{N}\left|\frac{{x}_{i}-{\widehat{x}}_{i}}{{x}_{i}}\right|$$

## Results and discussions

### Marshall stability

**Fig. 3:**
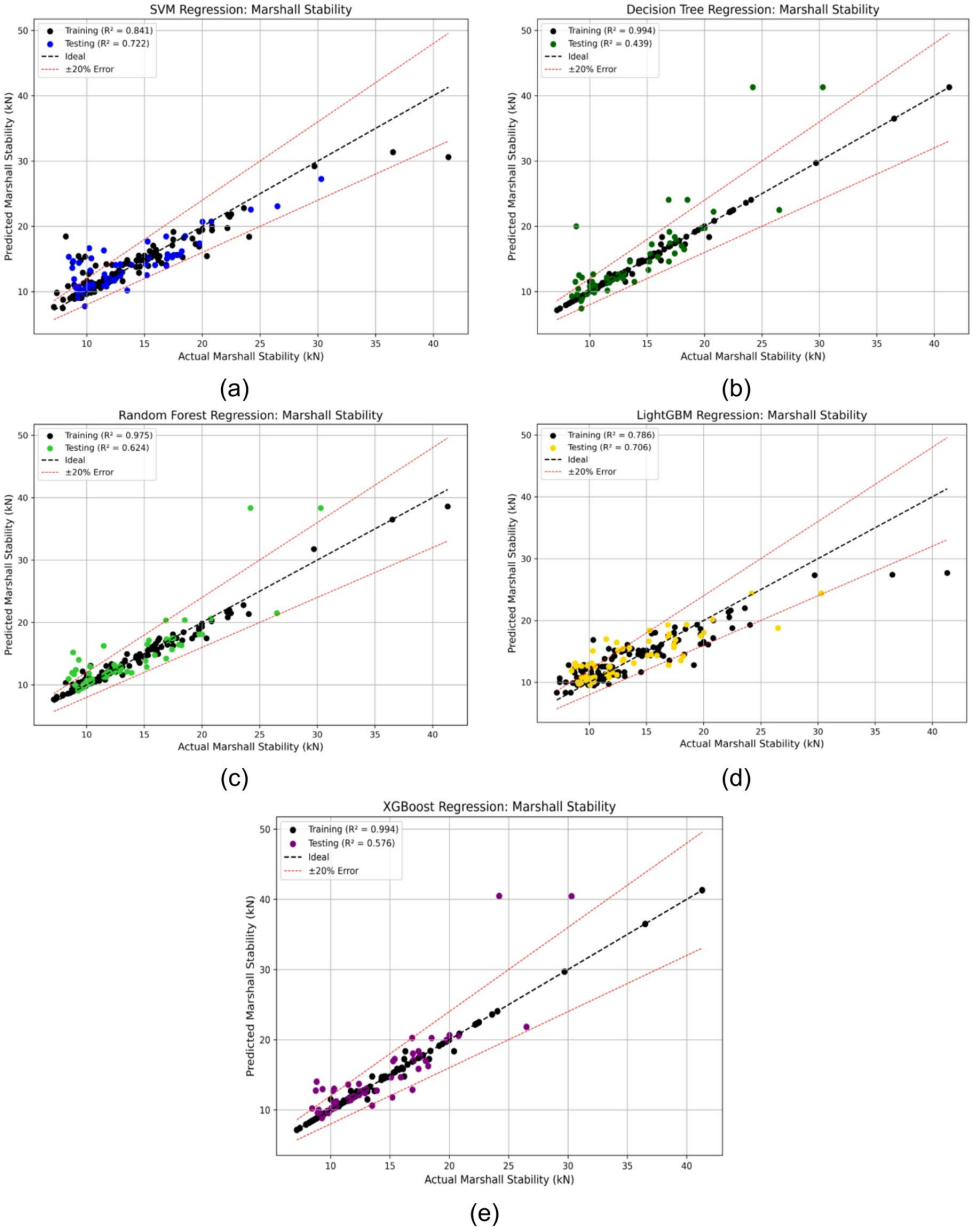
Regression plots for Marshall Stability using (**a**) SVM, (**b**) DT, (**c**) RF, (**d**) LGBM, and (**e**) XGB models showing training and testing performance.

To evaluate the performance of various machine learning models in predicting Marshall Stability, a series of regression and error comparison analyses were conducted. Figure 3 (a-e) presents the regression plots of five base models, SVM, DT, RF, LGBM, and XGB applied to the testing dataset. Among these, the Decision Tree and XGB models demonstrated perfect fit on training data (R^2^ = 0.994) but underperformed on the test data (DT: 0.439 and XGB: 0.576), suggesting overfitting. The SVM model showed more balanced performance, though with slightly lower accuracy.

To enhance model generalization and mitigate overfitting, PSO was employed to fine-tune the hyperparameters of LGBM and XGB, which are known for their strong predictive capabilities but high sensitivity to parameter tuning. These boosting models were chosen specifically for optimization due to their complex architectures and their tendency to overfit, as reflected by their near-perfect training performance and relatively lower test accuracy. Boosting algorithms inherently offers strong predictive power, and fine-tuning their complex hyperparameters (e.g., learning rate, depth, subsampling) is crucial for achieving optimal generalization^68,69^.

**Fig. 4:**
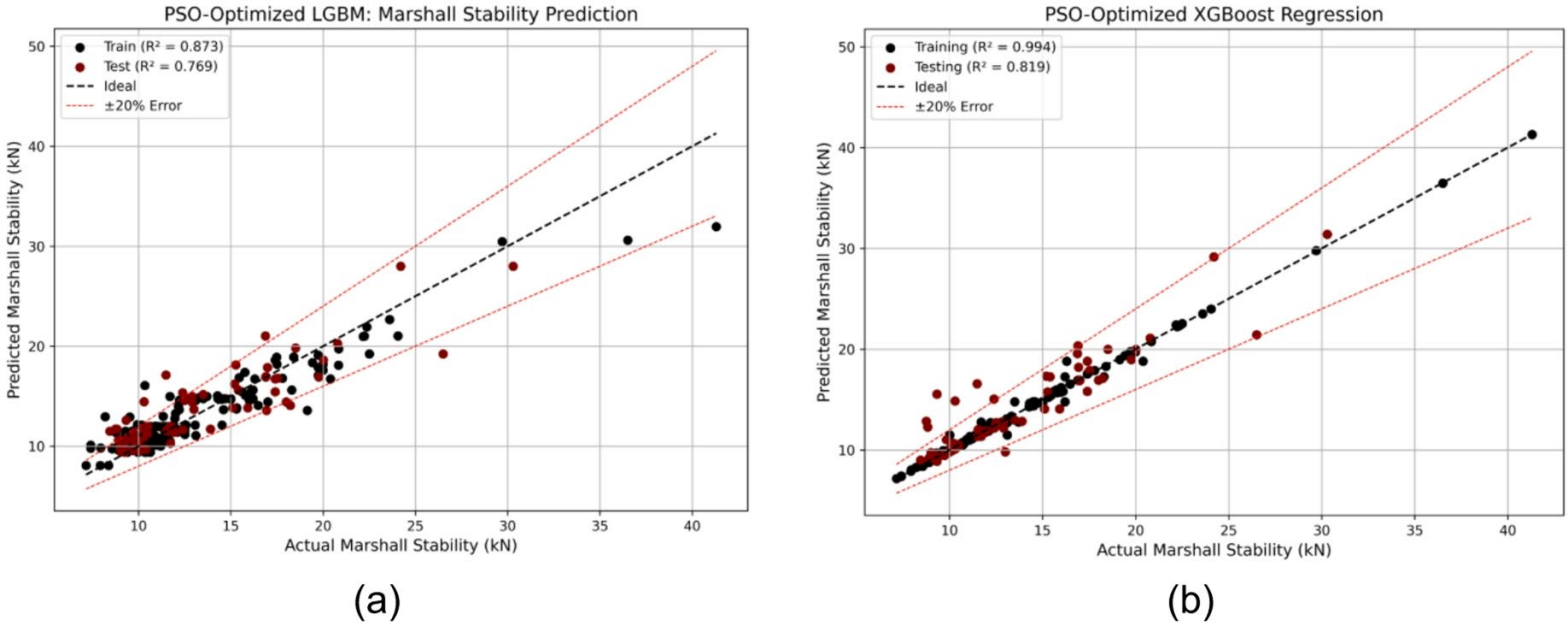
Regression plots for Marshall Stability using PSO-optimized models: (**a**) LGBM and (**b**) XGB, showing improved predictive alignment and reduced generalization error compared to base models.

Table 2 summarizes the optimal hyperparameters obtained via PSO. The performance gains from optimization are substantial. The test R² of LGBM improved from 0.706 to 0.769, while XGB improved from 0.576 to 0.819, representing relative increases of approximately 8.9% and 42.2%, respectively. As shown in Fig. 4 (a, b), the regression lines of the optimized models align more closely with the observed values, demonstrating more stable and accurate predictions. These results affirm the effectiveness of PSO in improving model generalization for Marshall Stability prediction^69,70^.

**Table 2:** Optimal hyperparameters of LGBM and XGB models obtained using PSO for Marshall stability prediction.

Parameters	PSO optimized LGB	PSO optimized XGB
Max depth	4	6
Learning rate	0.2588	0.2912
N estimators	59	428
Subsample	0.9654	0.6185
Colsample by tree	0.9982	0.5615

**Fig. 5:**
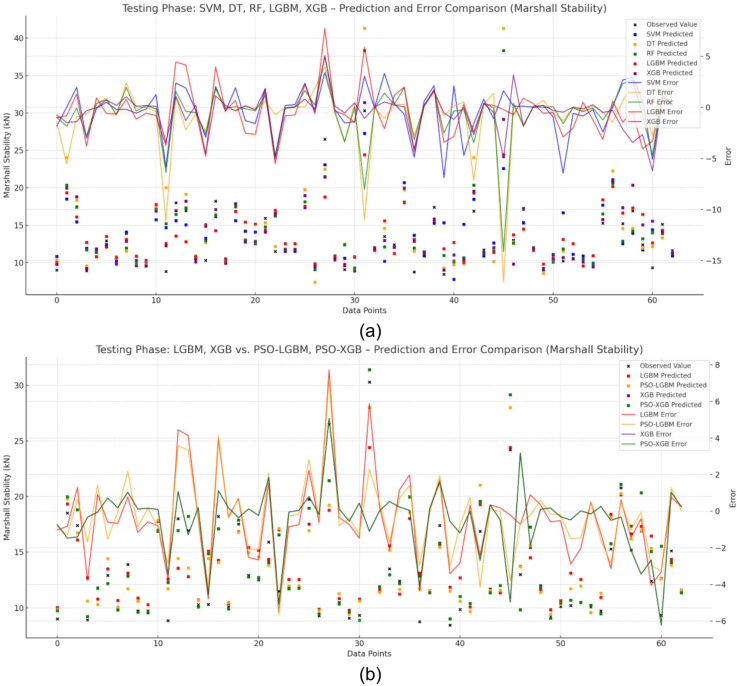
Error and prediction comparison for Marshall Stability using (**a**) base models (SVM, DT, RF, LGBM, XGB) and (**b**) PSO-optimized models, showing PSO-XGB’s closer alignment with observed values and reduced error fluctuations.

Figure 5a, b present a comprehensive comparison between the observed and predicted Marshall Stability values for all five base models, SVM, DT, RF, LGBM, and XGB along with the PSO-optimized LGBM and XGB models. The plots also include corresponding error lines, which allow for a clear visual assessment of each model’s predictive reliability and generalization behavior. Among the base models, SVM and RF demonstrated relatively consistent prediction trends with moderate errors, while DT and XGB exhibited signs of overfitting, as evidenced by larger error fluctuations on the test data.

In contrast, the PSO-optimized models particularly PSO-XGB produced predictions that were much closer to the observed values with substantially lower and smoother error lines, indicating enhanced accuracy and stability. This visual comparison reaffirms the effectiveness of PSO in addressing the overfitting issue seen in the base boosting models.

To quantitatively assess model performance, various evaluation metrics, R^2^, MSE, RMSE, MAE, and MAPE were computed for both training and testing datasets, as summarized in Table 3. As seen from the table, the PSO-XGB model again outperformed all others in terms of both accuracy and consistency.

**Table 3:** Comparison of evaluation metrics (R^2^, MSE, RMSE, MAE, MAPE) for training and testing phases across all models.

Models	SVM	DT	RF	LGB	XGB	PSO-LGB	PSO-XGB
R^2^ (Train)	0.841	0.994	0.975	0.786	0.994	0.873	0.994
R^2^ (Test)	0.722	0.439	0.624	0.706	0.576	0.769	0.819
MSE (Train)	4.069	0.152	0.652	5.479	0.152	3.254	0.166
MSE (Test)	5.645	11.405	7.642	5.987	8.613	4.705	3.685
RMSE (Train)	2.017	0.389	0.807	2.341	0.389	1.804	0.407
RMSE (Test)	2.376	3.377	2.764	2.447	2.935	2.169	1.920
MAE (Train)	1.148	0.128	0.533	1.579	0.129	1.285	0.194
MAE (Test)	1.679	1.660	1.535	1.793	1.455	1.581	1.177
MAPE (Train)	9.160	0.910	4.000	11.93	0.920	9.880	1.440
MAPE (Test)	14.17	11.61	11.10	14.00	9.860	11.89	9.030

### Marshall flow

**Fig. 6:**
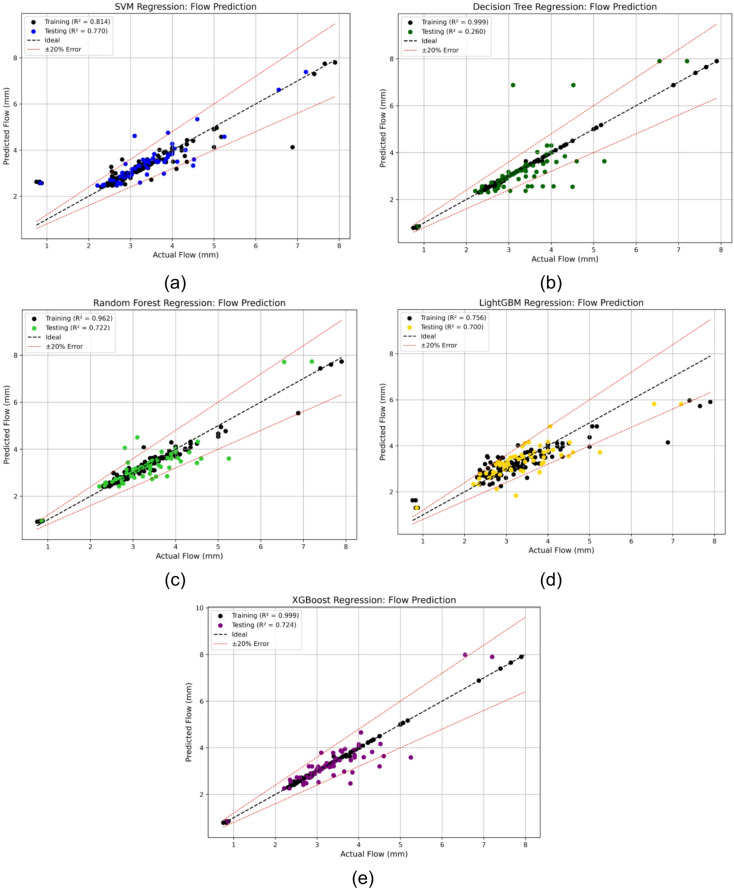
Regression plots for Marshall Flow using (**a**) SVM, (**b**) DT, (**c**) RF, (**d**) LGBM, and (**e**) XGB models showing training and testing performance.

A similar modelling approach to that used for Marshall Stability was adopted to predict Marshall Flow, ensuring consistency in model training, evaluation, and optimization across both performance indicators. Initially, five machine learning models, SVM, DT, RF, LGBM, and XGB were applied to the dataset. The regression plots for these base models, shown in Fig. 6 (a-e), highlight their predictive behavior on the testing set.

As observed in the stability prediction task, the DT and XGB models exhibited perfect or near-perfect performance on the training data but suffered from reduced generalization, as reflected in their lower test R^2^ scores and fluctuating error patterns. In contrast, the SVM and RF models showed more balanced performance across training and testing phases.

To enhance model generalization and reduce overfitting, PSO was again employed to optimize the hyperparameters of the LGBM and XGB models. These models were selected for tuning due to their complex boosting architectures, which are highly sensitive to hyperparameters such as learning rate, tree depth, and subsampling ratios. The optimal values obtained through PSO are presented in Table 4. The benefits of PSO optimization are clearly reflected in the test performance. The test R^2^ of LGBM improved from 0.700 to 0.762, and for XGB from 0.724 to 0.827, corresponding to relative improvements of 8.9% and 14.2%, respectively. These gains demonstrate the effectiveness of the optimization process in enhancing model accuracy for Marshall Flow prediction^69,70^.

**Table 4:** Optimal hyperparameters of LGBM and XGB models obtained using PSO for Marshall flow prediction.

Parameters	PSO optimized LGB	PSO optimized XGB
Max depth	9	6
Learning rate	0.1152	0.2967
N estimators	270	214
Subsample	0.8947	0.8224
Colsample by tree	0.6000	0.8884

**Fig. 7:**
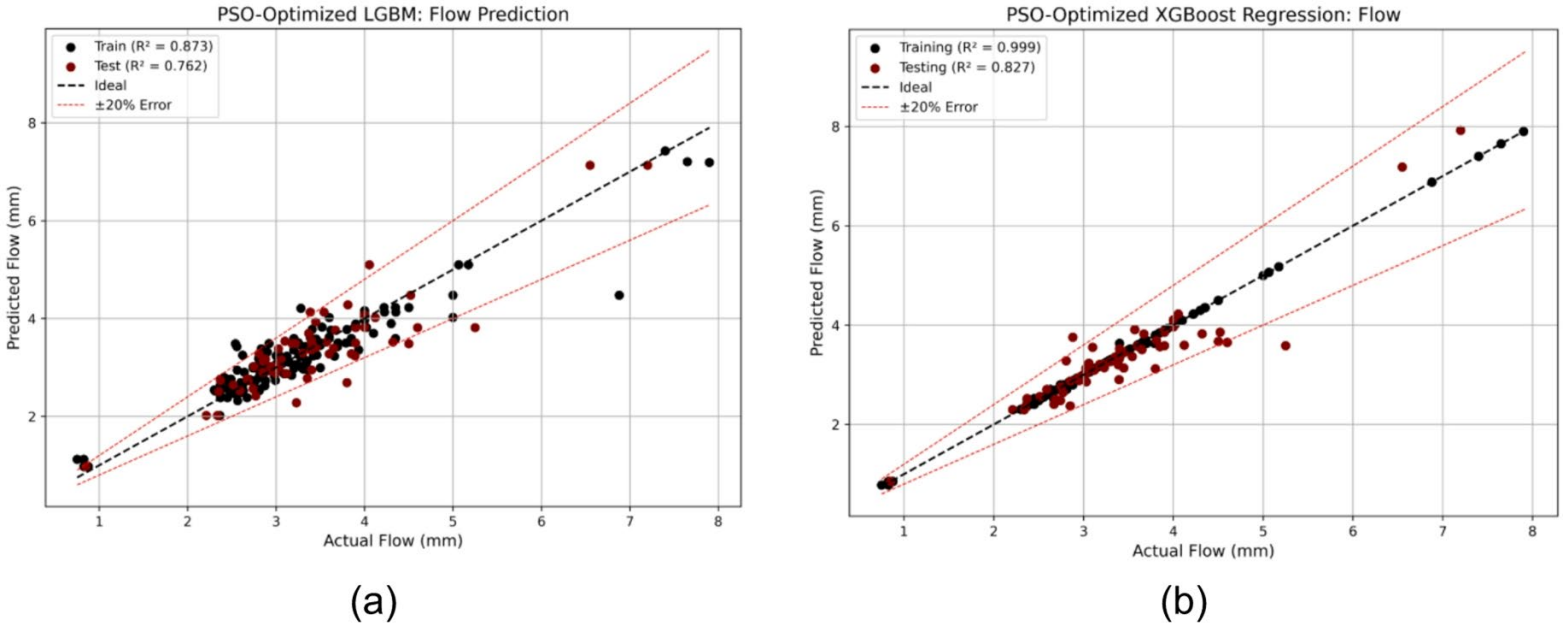
Regression plots for Marshall Flow using PSO-optimized models: (**a**) LGBM and (**b**) XGB, showing improved predictive alignment and reduced generalization error compared to base models.

The regression plots in Fig. 7 (a, b) illustrate the improved alignment between predicted and observed values for the optimized models. Notably, the PSO-XGB model closely follows the observed values with minimal dispersion, indicating high predictive accuracy.

**Fig. 8:**
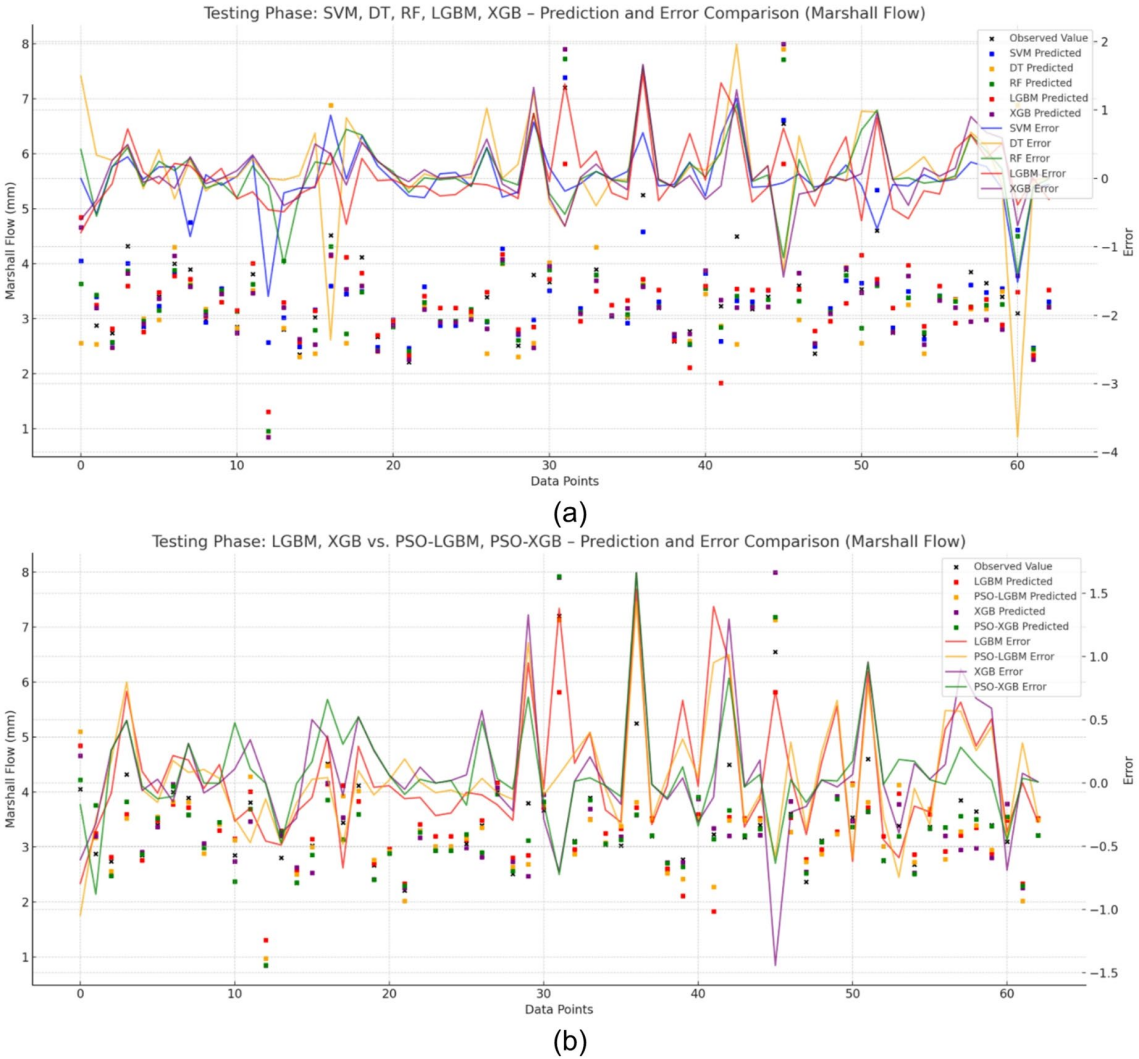
Error and prediction comparison for Marshall Flow using (**a**) base models (SVM, DT, RF, LGBM, XGB) and (**b**) PSO-optimized models, showing PSO-XGB’s closer alignment with observed values and reduced error fluctuations.

Further, Fig. 8a, b provide a comprehensive comparison of observed versus predicted values alongside error curves for all models. The PSO-optimized models, particularly PSO-XGB, exhibit lower error magnitudes and smoother trends compared to their base counterparts. On the other hand, models like DT and base XGB display larger and more erratic error profiles, reaffirming earlier observations of overfitting.

Finally, performance metrics including R^2^, MSE, RMSE, MAE, and MAPE were computed for both training and testing phases to provide a quantitative assessment of each model’s effectiveness. The results, summarized in Table 5, further confirm the superiority of the PSO-XGB model in terms of both accuracy and generalization.

**Table 5:** Comparison of evaluation metrics (R^2^, MSE, RMSE, MAE, MAPE) for training and testing phases across all models.

Models	SVM	DT	RF	LGB	XGB	PSO-LGB	PSO-XGB
R^2^ (train)	0.814	0.999	0.962	0.756	0.999	0.873	0.999
R^2^ (test)	0.770	0.260	0.722	0.700	0.724	0.762	0.827
MSE (train)	0.187	0.001	0.038	0.245	0.001	0.127	0.001
MSE (test)	0.196	0.629	0.236	0.256	0.235	0.203	0.147
RMSE (train)	0.433	0.036	0.194	0.495	0.036	0.357	0.037
RMSE (test)	0.443	0.793	0.486	0.506	0.485	0.450	0.383
MAE (train)	0.204	0.012	0.115	0.334	0.014	0.239	0.013
MAE (test)	0.274	0.438	0.309	0.374	0.309	0.326	0.233
MAPE (train)	10.38	0.50	3.67	11.39	0.56	7.83	0.53
MAPE (test)	10.20	11.62	8.60	10.92	8.10	9.37	6.16

### Taylor diagram analysis

To further evaluate the predictive performance and consistency of the applied machine learning models, Taylor diagrams were used as a comprehensive visual diagnostic tool. Taylor diagrams provide a compact representation of three statistical metrics simultaneously: the correlation coefficient (R), standard deviation, and the centered root mean square error (CRMSE) between predicted and observed values. These plots are widely employed in model validation due to their ability to simultaneously convey both the strength and variability of a model’s predictions relative to actual observations^60,71,72^.

**Fig. 9:**
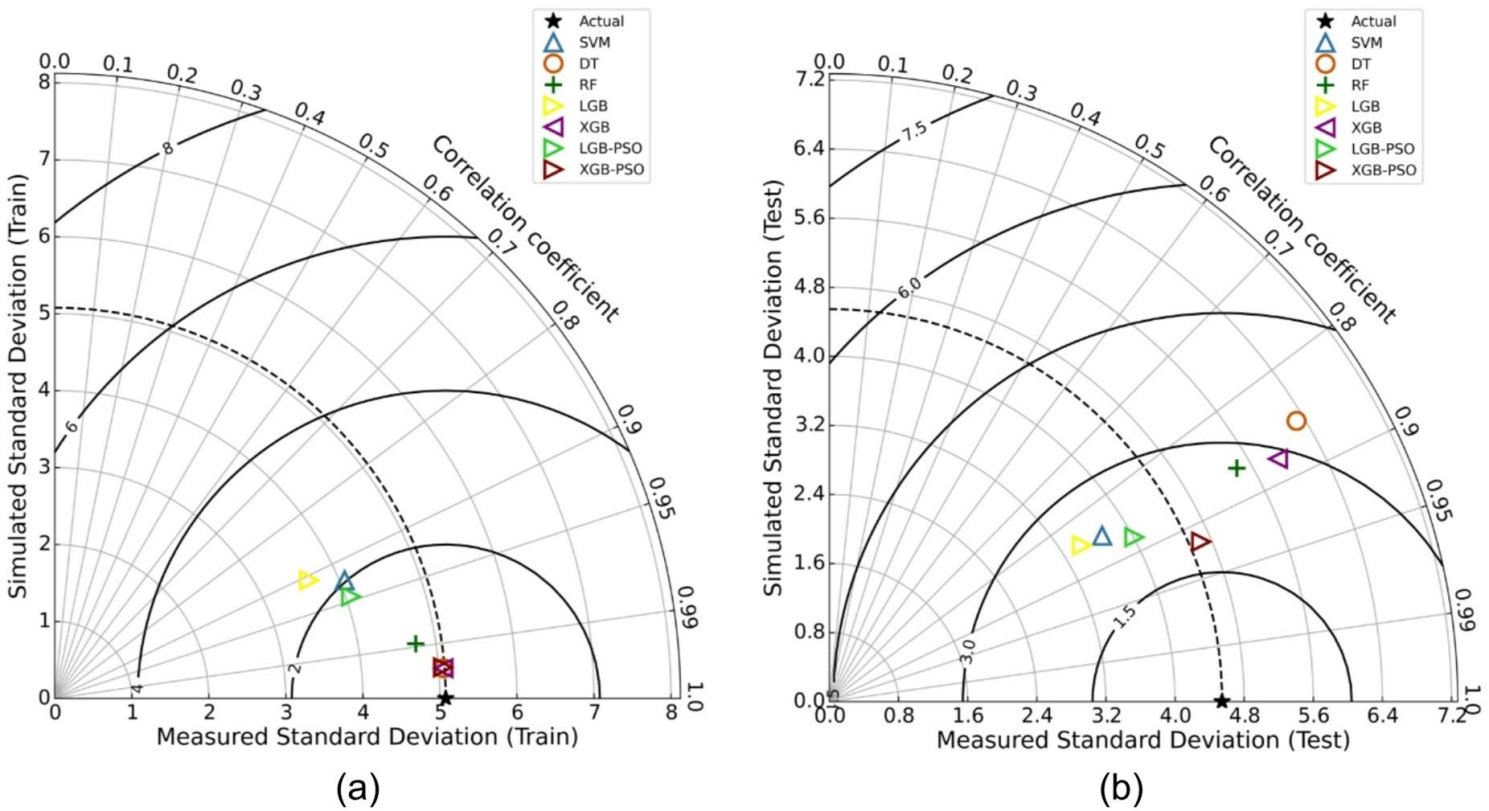
Taylor diagrams for Marshall Stability prediction showing performance of all models in (**a**) training and (**b**) testing phases.

The Taylor diagrams for Marshall Stability are shown in Fig. 9(a) for the training phase and Fig. 9(b) for the testing phase. In the training phase diagram, most models cluster near the reference point, indicating strong learning performance. Specifically, DT, XGB, and PSO-XGB exhibit very high correlations (*R* ≈ 0.99), and nearly identical standard deviations compared to the observed data. However, this close proximity to the ideal point is partially due to overfitting, particularly in the case of DT and base XGB. The generalization performance, as illustrated in Fig. 9(b), reveals clearer distinctions. The PSO-XGB model is located closest to the reference point with a correlation of 0.9185, nearly matching the standard deviation of the observed data. This indicates that the PSO-tuned XGB not only retained high correlation but also captured the variability in the test data most effectively. In contrast, models like DT and XGB without tuning exhibit greater deviations and lower correlations, further supporting the previously observed overfitting behavior. The SVM, RF, and PSO-LGBM models also demonstrate competitive performance, though slightly behind PSO-XGB in terms of proximity to the ideal point.

**Fig. 10:**
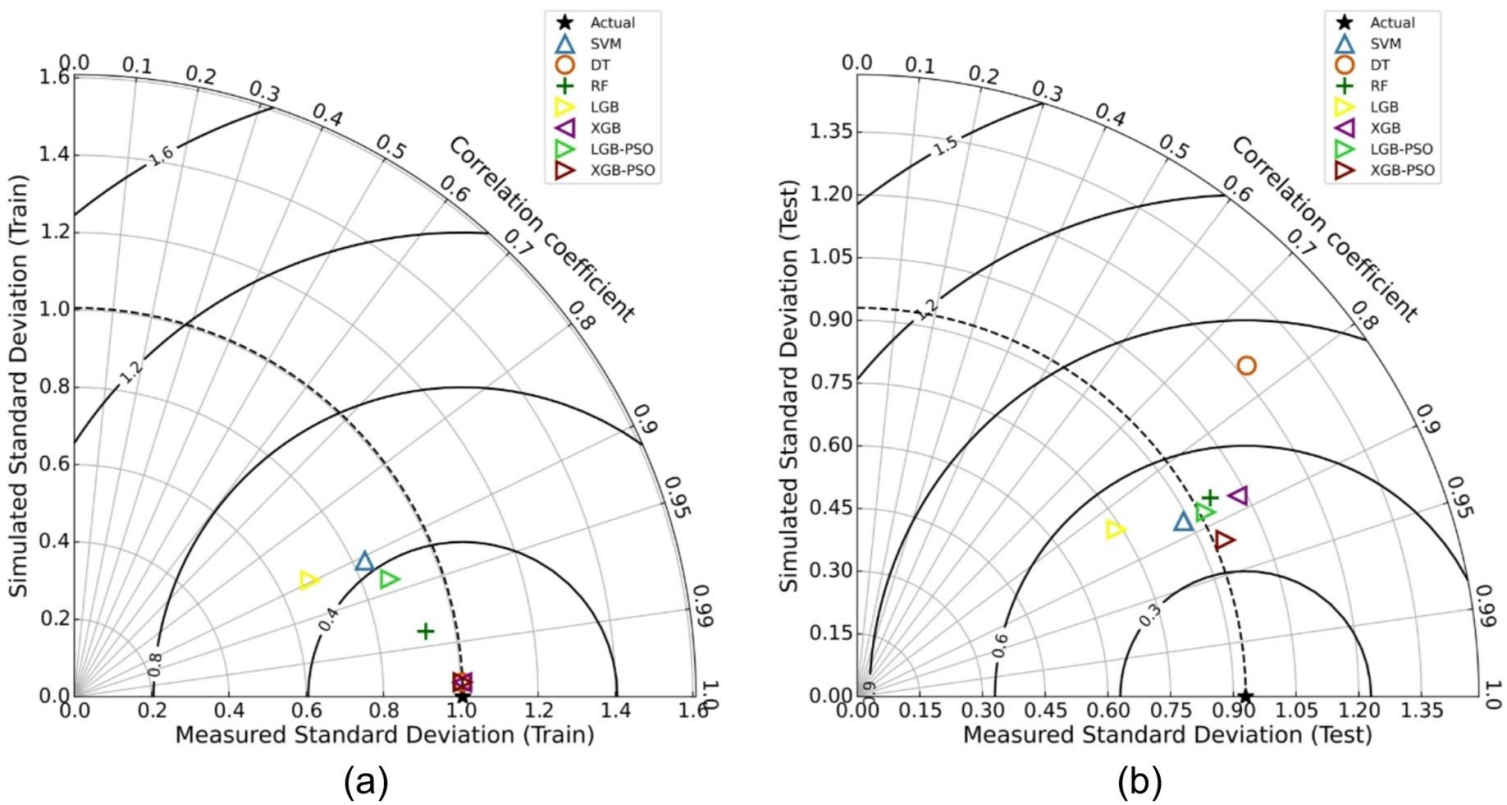
Taylor diagrams for Marshall Flow prediction showing performance of all models in (**a**) training and (**b**) testing phases.

A similar trend is observed in the Taylor diagrams for Marshall Flow, displayed in Fig. 10a (training) and Fig. 10b (testing). During training (Fig. 10a), several models including XGB, DT, and both PSO-optimized models appear to achieve near-perfect fits, with correlation coefficients nearing 0.99 and minimal CRMSE. Again, this suggests strong in-sample learning, but possibly at the expense of generalizability. The testing phase results (Fig. 10b) more accurately reflect model robustness. The PSO-XGB model maintains a high correlation of approximately 0.9201, while also closely aligning with the observed standard deviation of 0.9577. It is followed closely by PSO-LGBM and RF, which exhibit solid test performance. Meanwhile, DT and base XGB again deviate from the reference point, confirming their limited ability to generalize despite high training performance.

Across both performance indicators, Marshall Stability and Marshall Flow and both phases (training and testing), the PSO-optimized XGB model consistently exhibits the most favorable position in the Taylor diagrams.

### SHAP analysis

To further interpret the predictions made by the machine learning models particularly the PSO-optimized XGB model, which demonstrated superior performance, SHAP analysis was conducted. SHAP provides both global and local interpretability by assigning an importance value to each feature for a given prediction, grounded in cooperative game theory^60^. This makes it particularly valuable for understanding the inner mechanics of complex models like XGB^68^.

#### SHAP summary and feature importance

**Fig. 11:**
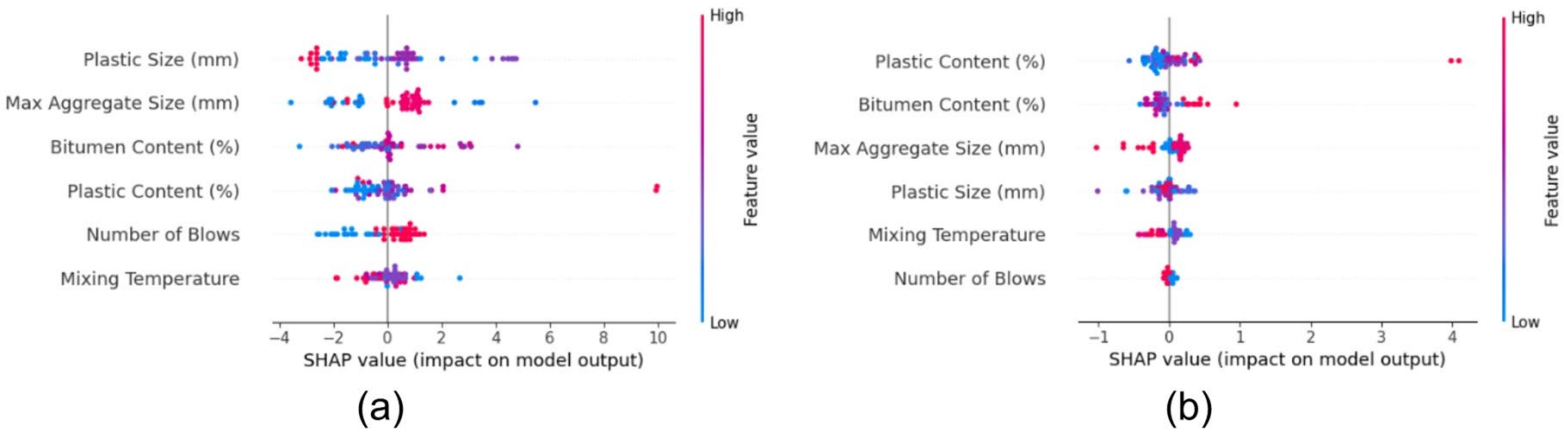
SHAP summary plots (**a**) Marshall stability (**b**) Marshall flow.

The SHAP summary plots, shown in Fig. 11, offer a global overview of feature influence by visualizing the impact, magnitude, and direction of each input variable on the model output. In the case of Marshall Stability (Fig. 11a), the most influential features identified were Plastic size, Max aggregate size, and Bitumen content. These variables not only contributed significantly to the model predictions but also exhibited clear nonlinear interactions, as shown by the varying SHAP value dispersions across feature ranges. For Marshall Flow (Fig. 11b), the dominant variables were Plastic content, Bitumen content, and Max aggregate size. This indicates that both the material composition and granular characteristics play vital roles in determining flow characteristics, highlighting the dual influence of plastic modifiers and aggregate structure^13,49,73^.

**Fig. 12:**
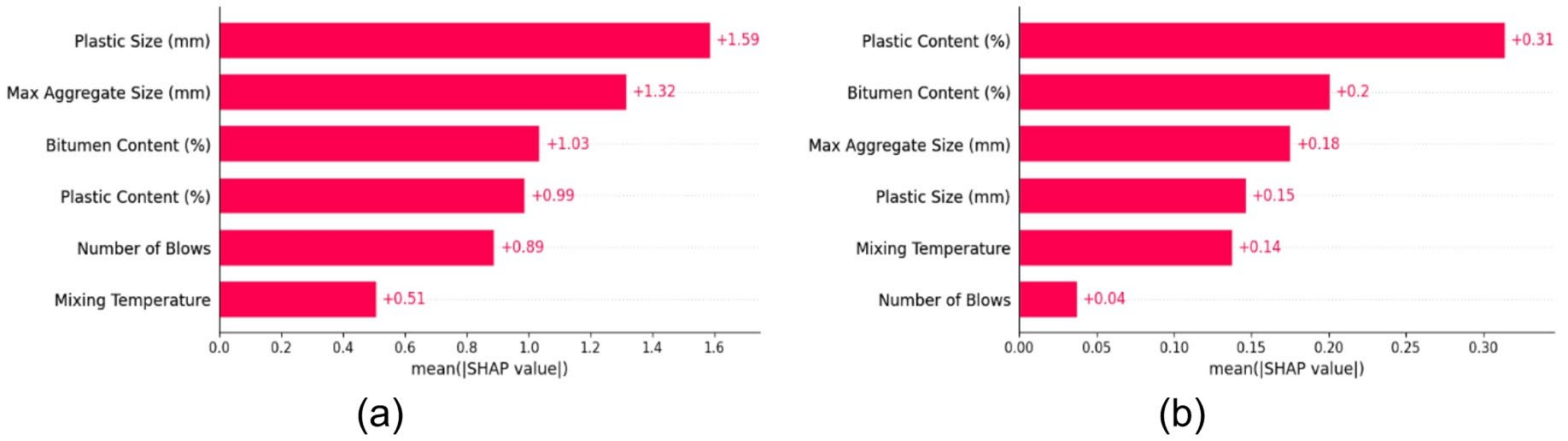
SHAP Feature Importance plots (**a**) Marshall stability (**b**) Marshall flow.

The SHAP feature importance bar plots, illustrated in Fig. 12, further quantify these insights by ranking the mean absolute SHAP values of all input features. For Marshall Stability (Fig. 12a), Plastic size had the highest importance with a mean SHAP value of approximately + 1.59, followed by Max aggregate size (+ 1.32) and Bitumen content (+ 1.03). This indicates that changes in Plastic size alone, on average, shift the model’s predicted stability by 1.59 kN, emphasizing its dominant influence.

In the case of Marshall Flow (Fig. 12b), Plastic content led the feature importance ranking with a mean SHAP value near + 0.31, followed closely by Bitumen content (+ 0.2) and Max aggregate size (+ 0.18). These values reflect the magnitude of impact these variables have on the predicted flow (in mm). Notably, plastic-related features (size or content) topped both rankings, confirming their crucial role in modifying asphalt mixture behavior.

#### SHAP dependence plots

To further understand how individual features affect predictions, SHAP dependence plots were generated for the top three influential features of each output.

**Fig. 13:**
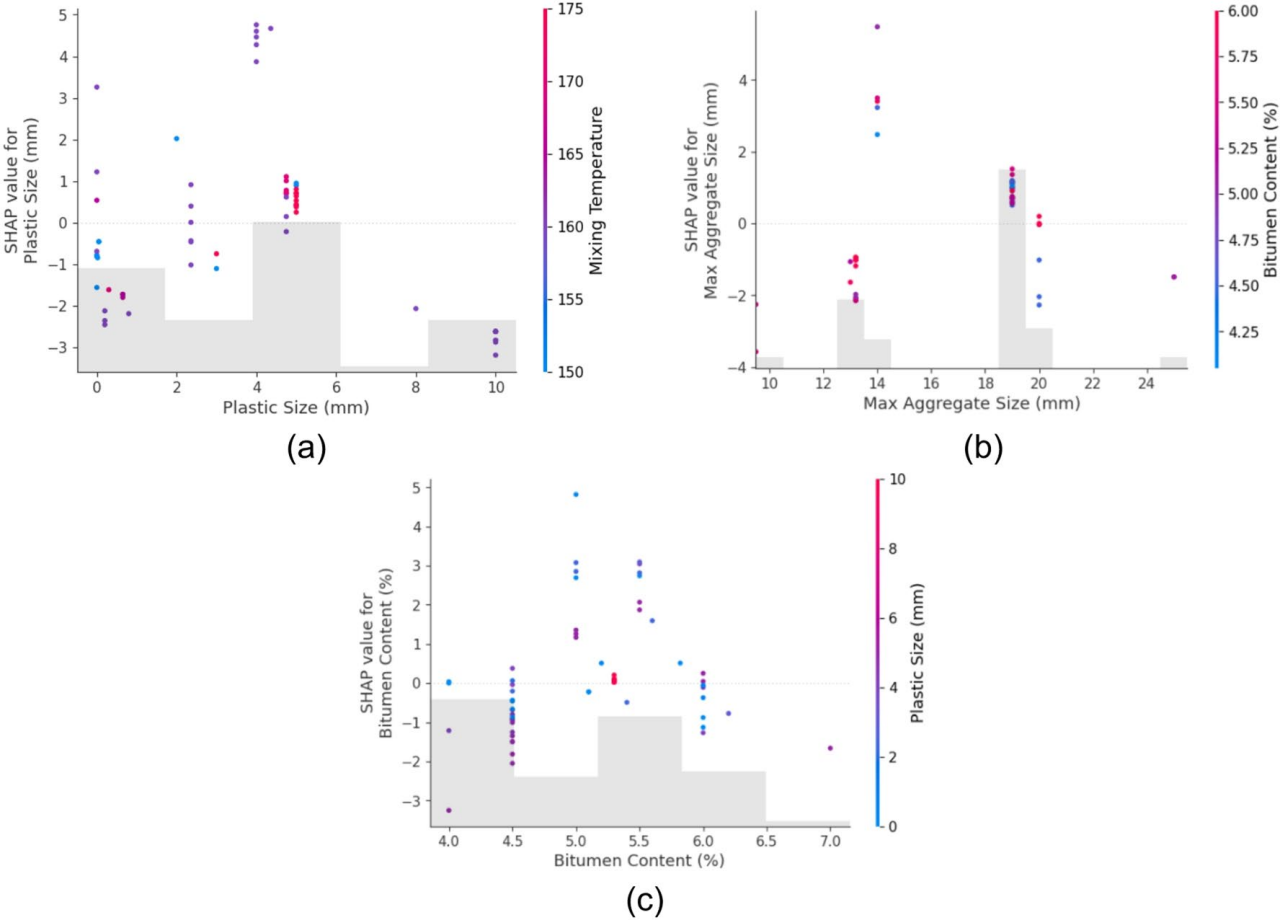
SHAP Marshall Stability top three feature dependence plots (**a**) Plastic size, (**b**) Max Agg Size, (**c**) Bitumen content.

For Marshall Stability (Fig. 13):

- Plastic size (Fig. 13(a)) shows a positive SHAP value trend at moderate sizes, indicating improved stability likely due to better interlocking or surface area effects.
- Max aggregate size (Fig. 13(b)) has a nonlinear contribution, where both very small and very large sizes reduce stability, likely due to poor gradation or packing density.
- Bitumen content (Fig. 13(c)) demonstrates a peak effect; too little or too much bitumen reduces stability, validating the optimal binder content principle in asphalt mix design.

**Fig. 14:**
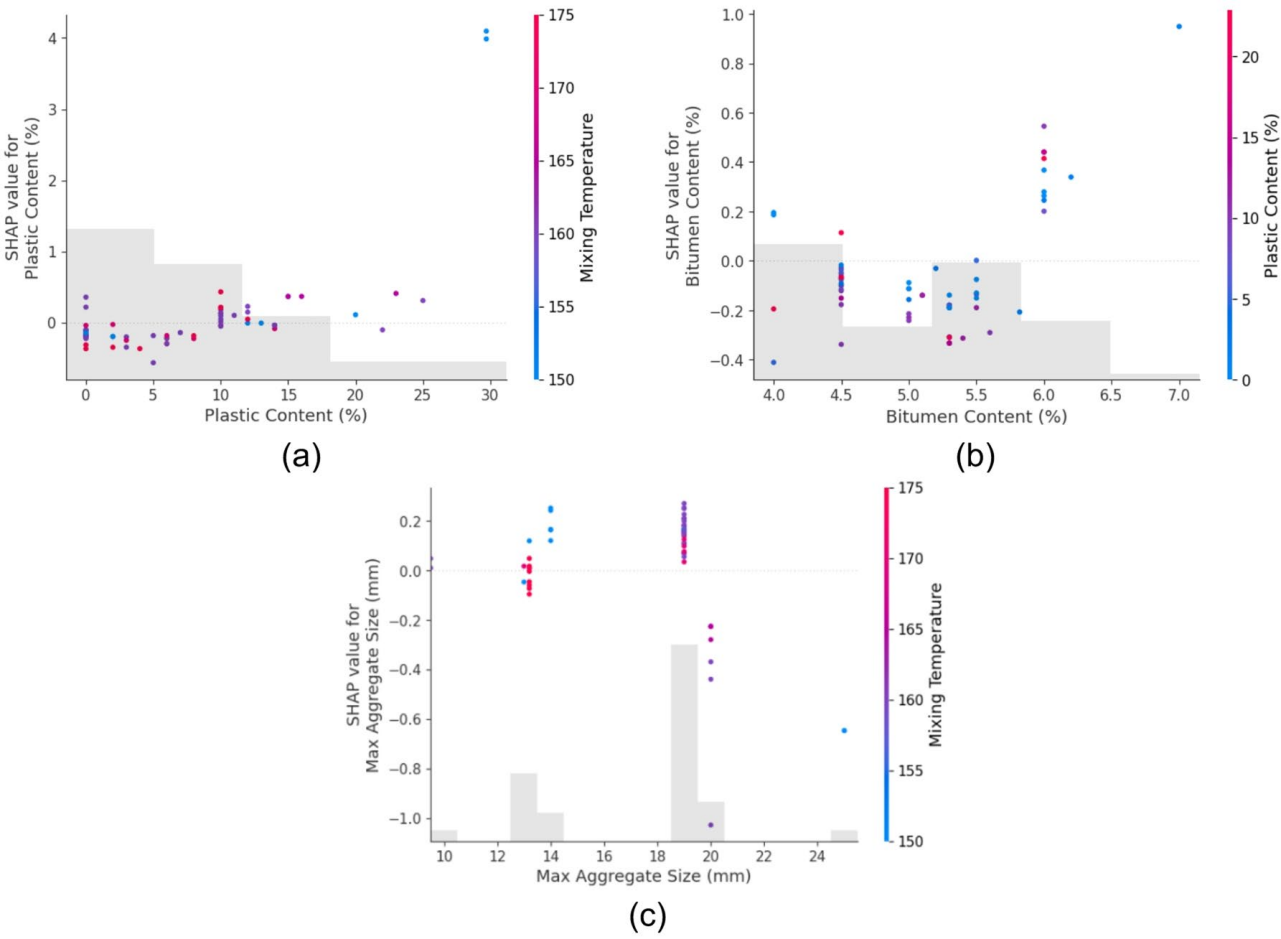
SHAP Marshall flow top three feature dependence plots (**a**) plastic content, (**b**) Bitumen content, (**c**) Max agg size.

In the case of Marshall Flow (Fig. 14):

- Plastic content (Fig. 14(a)) has a direct positive relationship with SHAP values, confirming that increasing plastic typically enhances flow, potentially due to its softening or lubricating effect.
- Bitumen content (Fig. 14(b)) also contributes positively up to a point, beyond which flow becomes excessive and potentially unstable.
- Max aggregate size (Fig. 14(c)) shows varied behavior, suggesting that while larger aggregates might reduce flow resistance, excessive size variation might lead to erratic flow behavior.

### Partial dependence plot analysis

To complement the global feature importance obtained through SHAP, PDPs were used to further investigate the average marginal effect of each feature on the predicted outputs. PDPs visualize how the model’s prediction changes as a single feature varies, while all other features are held constant. This helps to isolate and interpret the direct relationship between input variables and the model output, especially useful for complex models such as XGB^74,75^.

#### Marshall stability

**Fig. 15:**
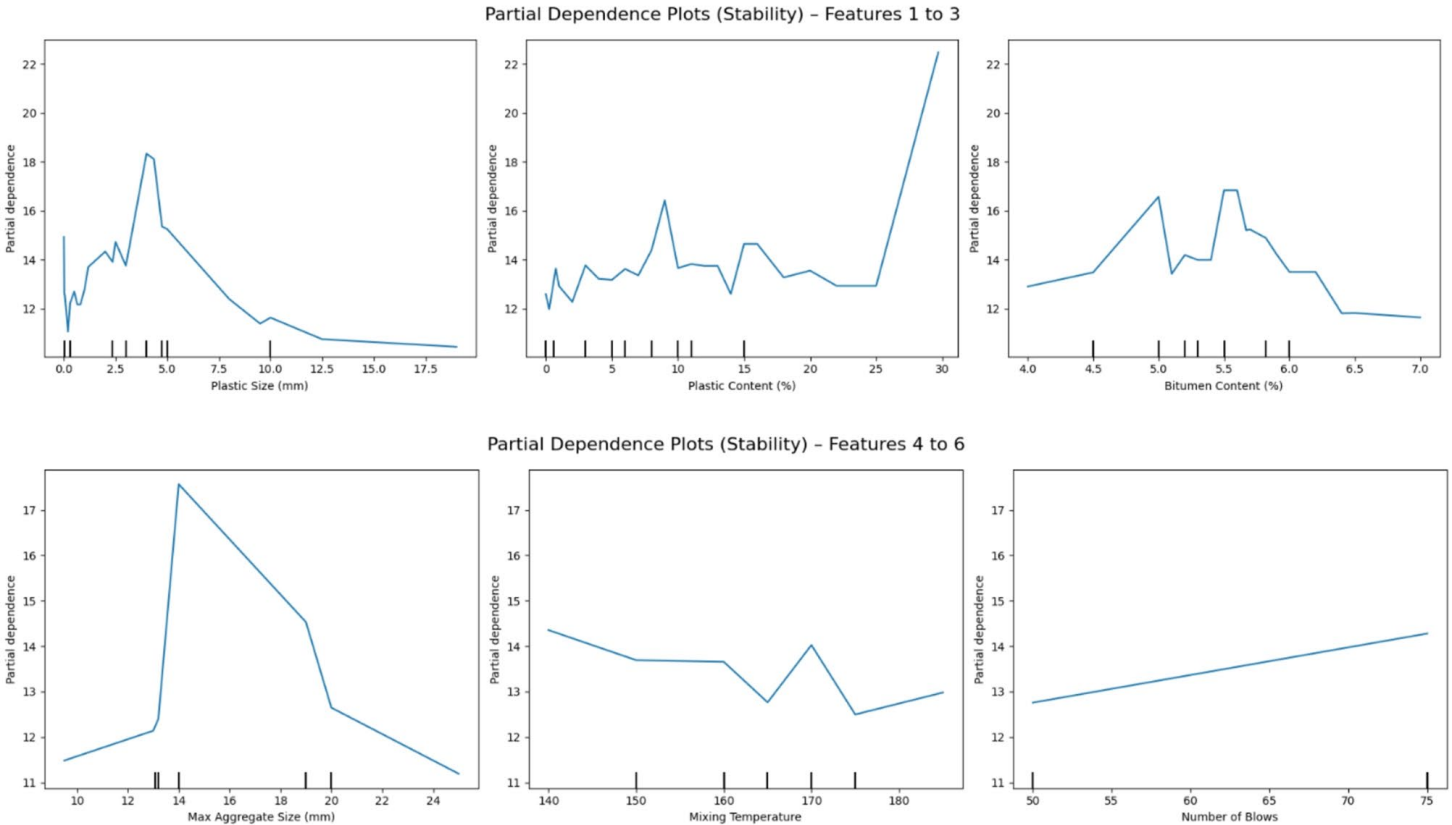
Partial dependence plots (PDP) for Marshall stability showing the marginal effect of input features: (top row) plastic size, plastic content, bitumen content; (bottom row) max aggregate size, mixing temperature, number of blows.

The PDPs for Marshall Stability are presented in Fig. 15, where each subplot illustrates the impact of a specific input feature on the predicted stability value. A clear trend is observed for Plastic size, which demonstrates a nonlinear positive influence, with stability increasing sharply up to an optimal size range before plateauing. This behavior suggests improved interlocking and surface area effects at moderate plastic sizes, consistent with the findings from SHAP dependence plots.

Similarly, Max aggregate size shows a convex pattern, where intermediate sizes improve stability, but both smaller and excessively large aggregates reduce it. This indicates an optimal gradation balance that enhances the structural skeleton of the asphalt matrix. Bitumen content, on the other hand, exhibits a parabolic relationship, with stability peaking around a mid-range bitumen value. This aligns with standard mix design theory, where too little binder leads to brittleness, and too much causes loss of stiffness^76^.

#### Marshall flow

**Fig. 16:**
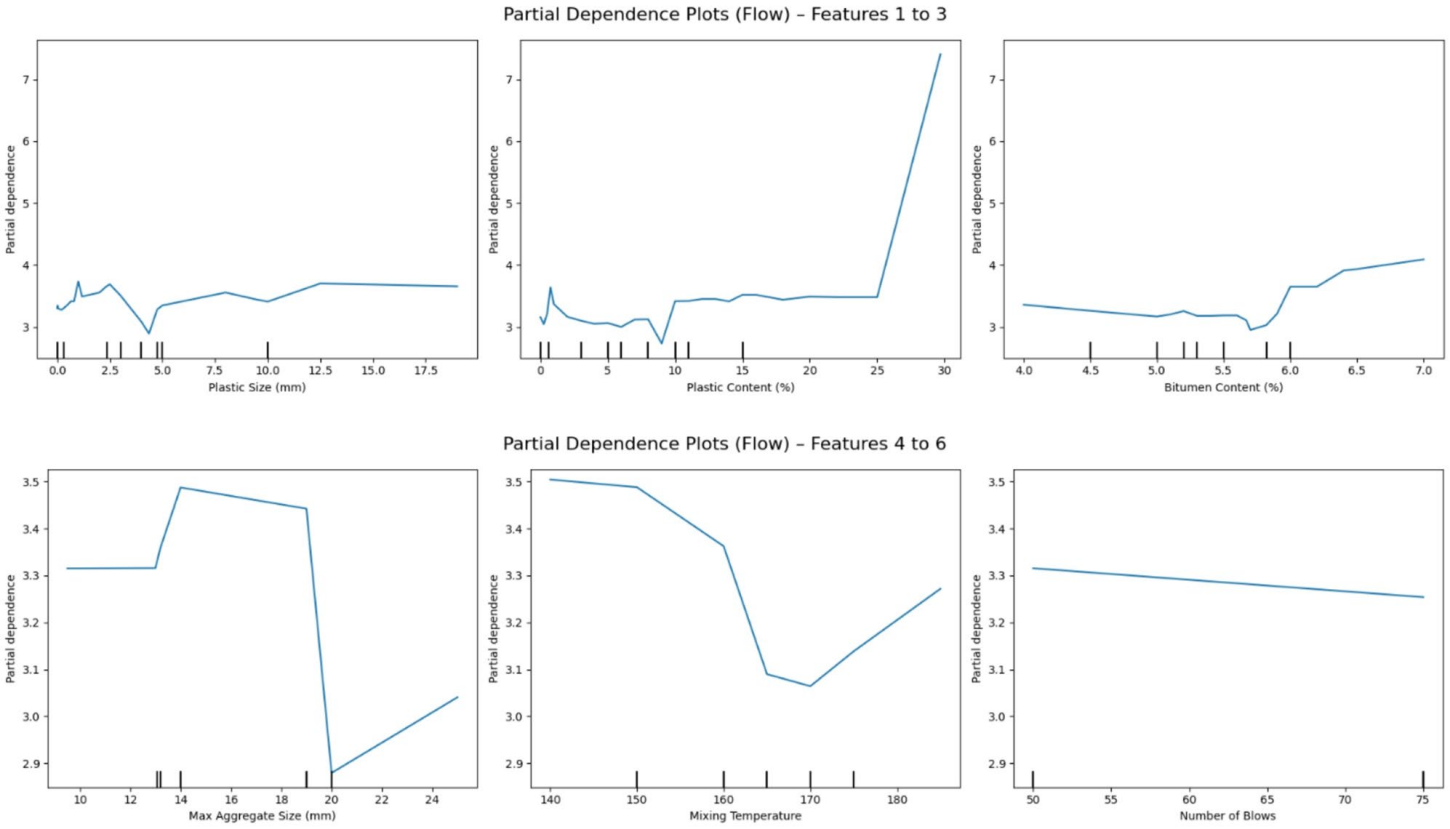
Partial dependence plots (PDP) for Marshall flow showing the marginal effect of input features: (top row) plastic size, plastic content, bitumen content; (bottom row) max aggregate size, mixing temperature, number of blows.

For Marshall Flow, the PDPs shown in Fig. 16 highlight different but equally meaningful patterns. Plastic content shows a strongly positive linear trend, indicating that increasing plastic content tends to increase flow, likely due to the softening effect plasticizers impart to the asphalt mix. This confirms the role of plastics in improving workability and deformation potential.

Bitumen content again displays a nonlinear influence, where flow increases steadily up to a certain content level, beyond which the curve flattens. This suggests a saturation point in binder effectiveness for flow enhancement. Max aggregate size reveals a mild negative trend beyond a certain threshold, indicating that larger aggregate sizes may slightly restrict flow due to increased interparticle resistance or reduced binder coating.

The PDP patterns thus provide a clear and interpretable global representation of feature effects and complement the SHAP dependence plots by showing average behavior across all samples. Together, they offer a robust explanation of how critical mix parameters affect both structural (stability) and deformation (flow) behavior in asphalt concrete.

### Individual conditional expectation analysis

To complement the global insights offered by SHAP and PDP, ICE plots were employed to reveal how predictions vary across different observations when a single feature changes. ICE plots visualize the instance-level effect of a variable while keeping all others fixed, thus uncovering heterogeneity in feature influence^77,78^. These are especially valuable for understanding whether feature impacts are consistent across the dataset or interact with other variables.

#### Marshall stability

**Fig. 17:**
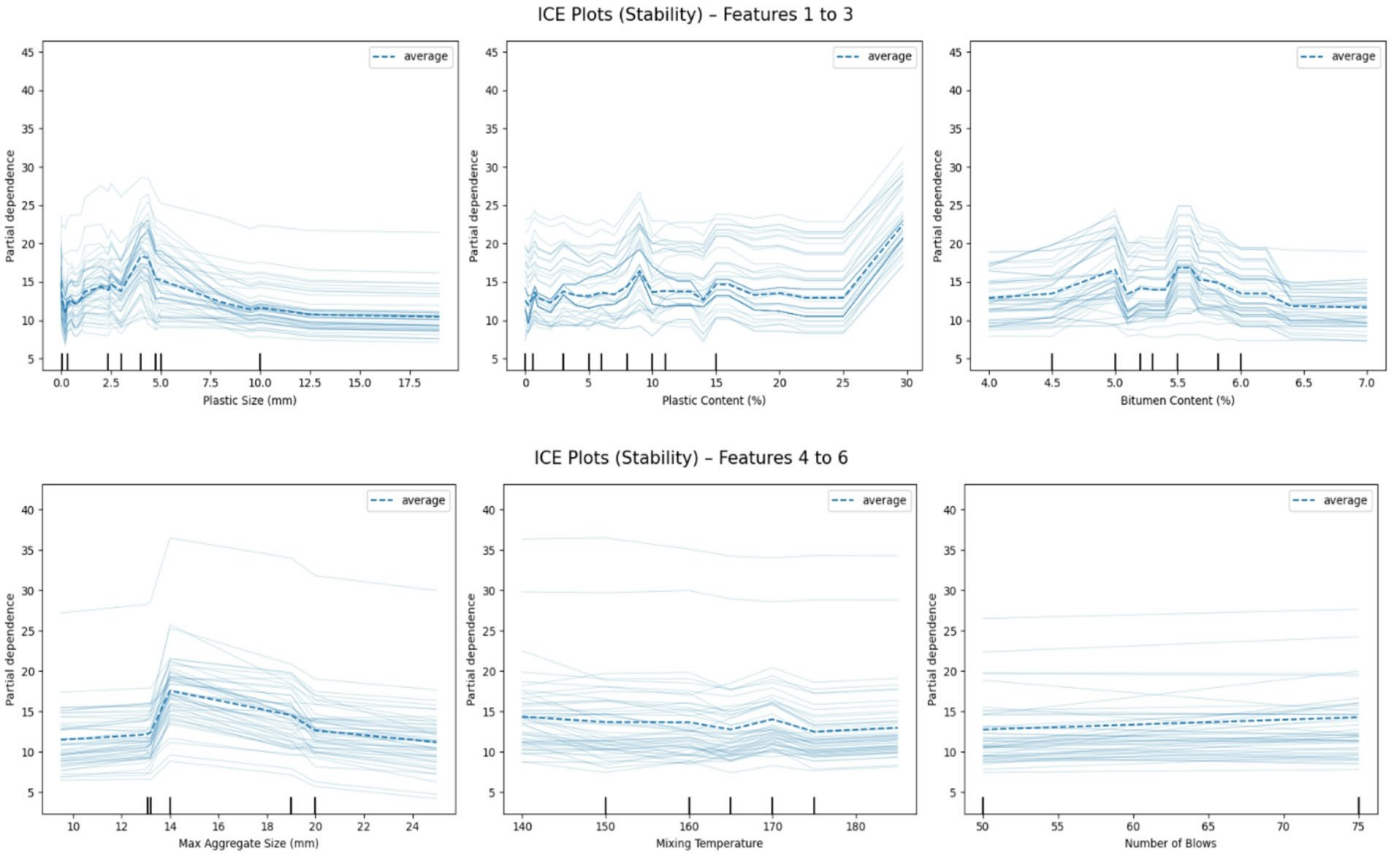
Individual conditional expectation (ICE) plots for Marshall stability showing sample-level prediction variations for: (top row) plastic size, plastic content, bitumen content; (bottom row) max aggregate size, mixing temperature, number of blows.

The ICE plots for Marshall Stability are presented in Fig. 17. In Fig. 17 (top row), the leftmost subplot illustrates the influence of Plastic Size (mm). A steep rise is observed in most ICE curves between 2.5 mm and 4.0 mm, indicating that many instances benefit from intermediate plastic sizes, with predicted stability increasing by up to 10–15 kN. This is followed by a flattening effect beyond 5 mm, showing diminishing marginal returns. This behavior suggests that moderate plastic particle sizes enhance structural interlocking, while excessively large particles contribute less effectively to load bearing. The middle subplot of Fig. 17 (top row) examines Plastic Content (%), which shows a bifurcated behavior. Below 15%, most ICE curves remain relatively flat, indicating a neutral effect on stability. However, beyond the 20% mark, many curves exhibit a sharp positive shift, with some instances showing predicted increases of 8–10 kN. This points to a threshold level where plastic additives begin contributing significantly to mix strength, likely through improved ductility or distribution of stress. In Fig. 17 (top row)-right, Bitumen Content (%) exhibits a parabolic trend across most ICE lines. Predicted stability peaks consistently around 5.3–5.5%, with values dropping slightly when the content moves outside this optimal range. In some instances, excessive bitumen beyond ~ 6% reduces predicted stability by ~ 2–4 kN, consistent with established Marshall mix behavior where stability peaks near the optimum asphalt content and declines at higher binder contents^79,80^.

Furthermore, in Fig. 17 (bottom row), the leftmost plot displays the behavior of Max Aggregate Size (mm). Here, most ICE curves rise noticeably between 14 mm and 18 mm, where stability gains reach up to 8–12 kN. Beyond 20 mm, however, the effect plateaus or even decreases, suggesting that excessively large aggregates may compromise packing density or lead to non-uniform stress distribution. In the center plot of Fig. 17 (bottom row), Mixing Temperature (°C) shows a much narrower influence. Across all ICE lines, predictions vary within a modest range of ± 2 kN, indicating a more stable and uniform contribution. Although higher temperatures can aid binder coating and workability, their direct impact on final stability appears to be relatively minor in this dataset. Finally, the rightmost plot in Fig. 17 (bottom row), presents Number of Blows, which reflects compaction effort. A slight upward trend is observed, with predicted stability increasing from approximately 12 kN to 14–15 kN as the number of blows rises from 50 to 75. The relatively linear nature of the trend suggests that compaction energy plays a consistent but moderate role in enhancing structural strength^81–83^.

#### Marshall flow

**Fig. 18:**
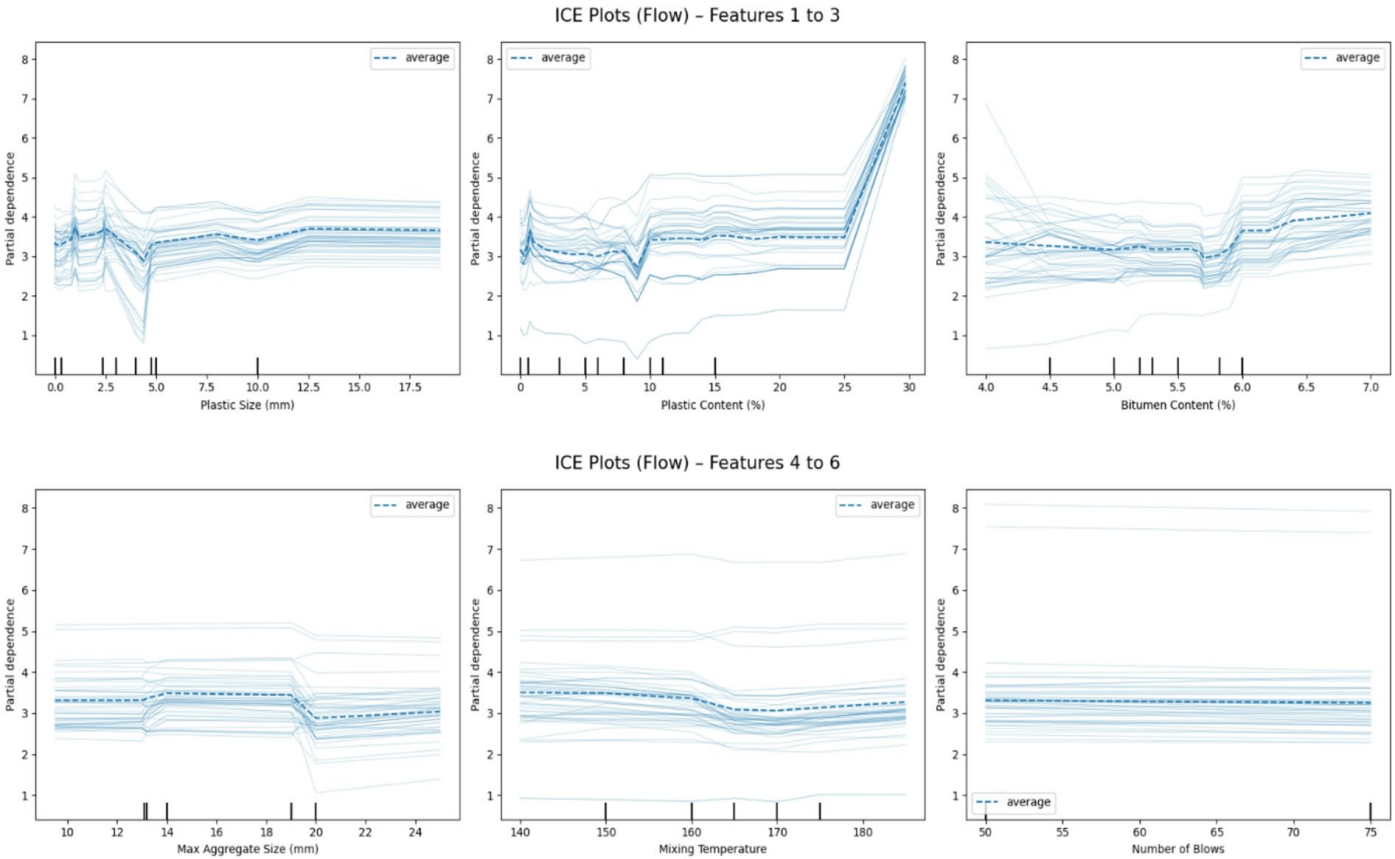
Individual conditional expectation (ICE) plots for Marshall flow showing sample-level prediction variations for: (top row) plastic size, plastic content, bitumen content; (bottom row) max aggregate size, mixing temperature, number of blows.

ICE plots for Marshall Flow are illustrated in Fig. 18. In Fig. 18 (top row), the left plot shows that Plastic Size (mm) produces only minor shifts in predicted flow, mostly within 1–1.5 mm across most ICE lines. A small trough appears around 5 mm, hinting at a potential interaction range where particle geometry minimally disrupts flow behavior. The center plot of Fig. 18 (top row) highlights Plastic Content (%), which emerges as a major influencing variable. At lower levels (0–10%), the effect is marginal. However, beyond 20%, a sharp increase is observed across several ICE curves, with predicted flow rising by 3–4 mm. This supports the notion that plastic significantly improves workability and deformability once a critical dosage is exceeded^9,11,18^. The right plot of Fig. 18 (top row) depicts Bitumen Content (%), where ICE lines display a steady positive trend. Most instances show a 1.5–2 mm increase in flow as bitumen content increases from 4% to 6.5%. A few curves level off after 6%, indicating that while binder content is crucial for flow, its effect plateaus after saturation.

In Fig. 18 (bottom row), the leftmost subplot explores Max Aggregate Size (mm). Here, ICE lines remain relatively flat with slight declines beyond 20 mm, particularly in samples with stiffer mixtures. This suggests that larger aggregate sizes may introduce interlocking resistance, slightly restricting the flow behavior of the asphalt. The middle plot of Fig. 18 (bottom row) demonstrates the effect of Mixing Temperature (°C), with a majority of ICE lines exhibiting very minor shifts typically within ± 0.5 mm. This implies that while temperature affects the ease of mixing and coating, its direct impact on post-compaction flow is limited in this scenario. Lastly, Number of Blows in Fig. 18 (bottom row)-right shows one of the most stable response profiles across the dataset. Most ICE curves follow a horizontal trend, indicating that changes in compaction energy from 50 to 75 blows have minimal influence on flow, with variation rarely exceeding 0.5 mm. This confirms that flow is predominantly driven by material characteristics rather than compaction effort.

Overall, the ICE plots reveal detailed and individualized trends that are consistent with the global results from SHAP and PDP but add depth by showcasing sample-specific variation. Features like plastic content, bitumen content, and max aggregate size showed the greatest heterogeneity, indicating strong interactions with other variables or threshold behaviors. In contrast, mixing temperature and number of blows demonstrated more uniform effects, underscoring their secondary roles in influencing both Marshall Stability and Flow.

## Comparative study

Recent studies have increasingly applied ML to predict MS and (MF of modified asphalt mixtures. A comparative summary of five recent ML-based studies and the current study is presented in Table 6.

Previous research utilized datasets of varying scales and origins. Kumar and Kumar^32^ examined plastic-modified asphalt using smaller laboratory datasets, identifying optimal plastic additive levels (e.g., ~ 7.5% PET) that significantly enhanced stability. Jalota and Suthar^84^ and Upadhya et al.^85^ compiled medium-sized datasets focused on fiber-modified asphalt mixes. Gul et al.^27^ employed an extensive dataset derived from general asphalt pavements across multiple road projects. In contrast, this study specifically focuses on plastic-modified asphalt mixtures with a sizable dataset (*N* = 210), providing targeted insights and improved predictive accuracy for mixes containing plastic additives.

Model complexity in prior works generally advanced from basic ensemble methods (Random Forest, ANN) with moderate accuracy (R^2^ ≈ 0.70–0.91) toward more sophisticated models like Multi-Expression Programming (MEP) used by Gul et al.^27^, achieving very high accuracy (R^2^ ≈ 0.97). The current research extends this by employing advanced gradient boosting (XGB, LGBM) with Particle Swarm Optimization (PSO), achieving exceptional predictive performance (R^2^ ≈ 0.82 for MS, ≈ 0.83 for MF), well within the results of previous studies specifically dealing with plastic additives.

While earlier studies primarily focused on Marshall Stability prediction, Gul et al.^27^ notably predicted both MS and MF, though for general asphalt mixtures. The current work similarly addresses both Stability and Flow, specifically within plastic-modified mixtures, highlighting the capability of ML to predict multiple critical properties simultaneously, which provide enhanced practical value for mix design optimization. Regarding interpretability, prior works typically conducted basic sensitivity analyses identifying crucial factors such as binder content or additive dosage. This study significantly advances interpretability by incorporating SHAP, PDP, ICE analyses, and Taylor diagrams, quantitatively revealing optimal plastic and bitumen content ranges and visually validating superior model generalization. These advancements offer deeper insights specifically tailored to plastic-modified asphalt, directly supporting improved decision-making in pavement engineering.

**Table 6:** Comparative summary of recent ML-based studies for Marshall stability prediction, highlighting dataset characteristics, models used, best-performing methods, and key insights.

Author/year	Dataset details	ML models used	Target variable (s)	Best model and performance	Key insights
^33^/ 2025	*N* = 265; inputs (5): bitumen content, plastic content, plastic size, bitumen grade	XGB, RF, Gradient Boosting, Bagging Regressor	Marshall Stability	**XGB -** Highest R (> 0.95 train, > 0.84 test), lowest MSE, RMSE, and MAPE values	XGB achieved top accuracy; SHAP showed plastic size and bitumen content as key features.
^32^/ 2023	Asphalt concrete with plastic waste; inputs (4) varying bitumen content (4–6%), plastic content, plastic size, binder grade (VG). *N* = 92	RF, Random Tree, bagging (RF, RT), ANN	Marshall Stability (MS)	**Random Forest** – Best *R* = 0.94 train, 0.89 test, RMSE ≈ 2.22 kN on test set.	RF outperformed other soft-computing models; sensitivity analysis found plastic particle size most influential on MS.
^84^/ 2023	*N* = 138 (PP fiber–reinforced asphalt); inputs: fiber content, fiber length, bitumen content, etc.	MLR, REP Tree, Random Tree, M5P (pruned & unpruned), RF	Marshall Stability (fiber-reinforced mix)	**Random Forest** – *R* ≈ 0.914, WI ≈ 0.95, RMSE ≈ 1.66 (testing).	RF performed best; bitumen content is most influential (sensitivity); ensemble trees effective for fiber-modified mixes.
^85^/ 2022	Laboratory study on hybrid fiber-modified asphalt (glass and carbon fibers); inputs (10): 5 mix types (GF, CF, and 3 GF-CF blends) × varying binder contents. *N* = 164.	ANN, Gaussian Process (PUK kernel), M5P model tree, Random Tree, MLR	Marshall Stability (hybrid fiber mix)	**ANN** – R^2^ ≈ 0.70, CC ≈ 0.84; MAE ~ 1.50 kN, RMSE ~ 1.83 kN on test data.	ANN slightly outperformed others; CF diameter and binder content key; moderate accuracy due to limited data.
^27^/ 2022	343 data points from 25 road projects (field cores and lab Marshall tests); inputs (8): mix design parameters (aggregate gradation, binder % 60/70, etc.).	ANN, ANFIS (neuro-fuzzy), MEP (gene-expression programming)	Marshall Stability and Flow	**MEP model** –, *R* = 0.968 (MS), 0.978 (MF). All models had *R* > 0.90 for MS & MF.	MEP outperformed ANN/ANFIS with explicit MS/MF equations; sensitivity confirmed trends; AI models reduce need for extensive lab trials.
Current Study 2025	Modified asphalt mixes with plastic waste and binder contents; inputs (6), *N* = 210.	SVM, DT, RF, LGBM, XGB; optimized via PSO (for XGB/LGBM).	Marshall Stability and Flow	**XGB + PSO** – best model; R^2^ ≈ 0.82 (MS), 0.83 (MF); lowest MAE/RMSE among models.	Boosting with PSO optimization achieved highest accuracy; SHAP/PDP/ICE identified key features; Taylor diagrams validated superior model generalization.

## Practical implications

The outcomes of this research have several practical implications for pavement engineering and infrastructure sustainability thus connecting the predictive insights of this study to real world road safety improvements, environmental benefits, and cost reductions.

### Enhanced road safety

Accurate prediction of MS and MF enables the design of mixtures that are both strong and flexible, reducing the risk of rutting and cracking under traffic loads. ICE analysis revealed that stability improved by 10–15 kN at optimal plastic sizes of 2.5–4 mm and increased by 8–10 kN beyond 20% plastic content, indicating that properly engineered plastic-modified asphalt can resist higher loads without premature failure. Flow values remained within 3–4 mm, ensuring sufficient flexibility to prevent thermal cracking^50,86^. These characteristics directly translate into improved road safety and longer-lasting pavements, with fewer failures and reduced maintenance needs.

### Sustainability and environmental benefits

Integrating 7.5–15% recycled plastic content into asphalt mixtures diverts substantial waste from landfills and mitigates environmental pollution^87,88^. The study highlights that flow increased by 3–4 mm above 20% plastic content, confirming workability at higher recycled content levels. By partially replacing virgin polymer modifiers, this approach supports circular economy principles, lowers the carbon footprint of pavement construction, and contributes to global efforts to manage plastic waste responsibly.

### Economic and construction efficiency

The optimized mixtures, guided by ML predictions and validated through PSO-XGB modeling, reduce the need for trial-and-error laboratory testing, saving time and material costs in the mix design stage. Optimal binder content around 5.3–5.5% and aggregate sizes of 14–18 mm provides a balance between strength and flexibility, potentially lowering lifecycle costs by reducing early maintenance interventions. Collectively, these findings support cost-effective, sustainable pavement construction suitable for large-scale implementation.

While recycled plastics can improve rutting resistance, durability and moisture performance, practical challenges such as dispersion, binder-plastic compatibility, and potential low-temperature brittleness^15^, must be actively managed. In practice, dispersion/compatibility are improved via wet-process high-shear blending and compatibilizers such as maleic-anhydride-grafted polyolefins (PE-g-MA/PP-g-MA), which enhance interfacial bonding and storage stability^19,89^. Sulfur (and polyphosphoric acid) crosslinking likewise reduces phase separation and strengthens the polymer-bitumen network^90^. In parallel, moisture susceptibility can be alleviated by plastic-powder aggregate coatings, which increase surface hydrophobicity and improve adhesion under wet conditions^14,91^.

Low-temperature brittleness noted for some plastomers (e.g., PE/PP) can be mitigated by hybrid modification such as blending plastics with elastomers (SBS, crumb rubber) and bio-oils which restores flexibility while retaining high-temperature stability. Recent studies and reviews report that SBS/CR-plastic hybrids and plastic plus bio-oil systems improve low-temperature or anti-fatigue performance without sacrificing rutting resistance when appropriately dosed^92,93^. Taken together, these standard mitigations enable practitioners to capture the net benefits (higher stability, better moisture resistance, and durable performance) documented for plastic-modified asphalt, provided that particle size, dosage, and mixing/compatibilization are specified and verified in QC (e.g., storage-stability checks, MSCR/BBR)^94,95^.

## Critical evaluation, future recommendations and limitations

### Critical evaluation of findings

Machine learning contributes by capturing the nonlinear, interacting effects among mixture variables (plastic content/size, binder content, aggregate size, temperature, compaction) on Marshall responses. Gradient-boosted trees (XGB/LGBM) assemble many shallow learners, enabling flexible response surfaces, while PSO searches the hyperparameter space (depth, learning rate, estimators, subsampling) to curb over/under-fitting and maximize out-of-sample accuracy. In our results, PSO-XGB increased test R^2^ for MS from 0.576→0.819 and reduced RMSE from 2.935→1.920; for MF, test R^2^ improved 0.724→0.827 with RMSE 0.485→0.383. These gains indicate that metaheuristic tuning materially improves generalization beyond untuned boosting for MS and MF of plastic-modified asphalt mixtures. However, a critical evaluation is necessary to understand the practical relevance and transferability of these results to real-world pavement engineering applications. Although the models achieved high predictive accuracy under laboratory conditions, field pavements are subject to dynamic traffic loads, moisture ingress, temperature fluctuations, and long-term material aging, which may alter the expected performance. For example, ICE analysis revealed that stability improved by 10–15 kN at optimal plastic particle sizes of 2.5–4 mm and by 8–10 kN when plastic content exceeded 20%. While these gains are significant in a controlled laboratory environment, actual roadways may experience differential settlements and repeated loading cycles that could influence the durability of these improvements. Similarly, flow values increasing by 3–4 mm above 20% plastic content suggest enhanced flexibility, but excessive deformation under heavy traffic could still occur in the field if mix design is not carefully validated. To verify generalizability, future work should validate the models on field sections representing different traffic categories (light/medium/heavy) and climates. Candidate targets include rutting depth progression, crack density, and ride quality (IRI), derived from periodic surveys and FWD back-calculated moduli. Model transfer will be assessed via external hold-out sites, temporal split validation, and calibration curves (predicted vs. observed), with thresholds for acceptable error set against agency specs. This protocol will confirm whether the MS/MF-trained models remain reliable under real construction variability and in-service aging.

Furthermore, the generalizability of the predictive models is limited to the input ranges considered in this study: plastic content (0–30%), bitumen content (4–7%), and maximum aggregate size (9.5–25 mm). Mixtures outside these ranges or subjected to unconventional traffic or climatic conditions would require additional model retraining or field verification to ensure reliable performance. Nonetheless, the combination of machine learning and optimization demonstrates strong potential to reduce experimental trials, guide performance-based mix design, and enhance decision-making in pavement engineering. These findings, when combined with field validation can support the adoption of safer, more sustainable, and performance-driven pavement construction practices.

### Future recommendations

The outcomes of this study demonstrate robust and accurate predictions for Marshall Stability and Flow using optimized gradient boosting techniques. However, several avenues remain open for further research.

- Expand the dataset by including the field performance data under varying traffic and environmental conditions, such as heavy-load traffic, temperature fluctuations, and moisture effect to improve the generalizability of the models for practical pavement applications.
- Expand performance modeling to include rutting resistance, fatigue cracking resistance, and moisture susceptibility, which are critical for long-term pavement durability.
- Design and execute a structured field-validation campaign to assess model transferability under real traffic and climate.
- Include additional input variables such as plastic type (e.g., PET, PE, PP) and mixing method (dry or wet) to capture their influence on performance and enhance the model’s field applicability.
- Explore advanced and hybrid AI models (e.g., deep learning or ensemble hybrids) to further enhance prediction accuracy and capture complex nonlinear interactions.
- Employ automated machine learning (AutoML) frameworks to streamline hyperparameter tuning and model selection processes.
- Investigate other sustainable additives, such as crumb rubber, bio-binders, or industrial by-products, alongside plastic waste to evaluate synergistic effects.
- Incorporate multi-objective optimization techniques to simultaneously consider strength, durability, cost-efficiency, and environmental impact in mix design.
- Develop user-friendly decision support tools based on trained models to assist engineers in real-time pavement material design.

### Limitations

Despite the strengths, this study has limitations. Primarily, the current dataset predominantly consists of laboratory-derived data, which may limit the direct transferability of the findings to field conditions. Additionally, while interpretability tools provided detailed insights, they remain inherently model-dependent, meaning further validation through controlled experimental trials or field validation would enhance confidence in practical applications. Moreover, although plastic type information was available in the raw dataset, it was not incorporated into the models to maintain simplicity and avoid overfitting with a limited sample size; future studies with larger datasets can explore its impact.

## Conclusions

This research developed reliable predictive models for MS and MF of plastic-modified asphalt mixtures using advanced machine learning methods specifically XGB and LGBM, optimized through PSO. Model performance and predictions were rigorously interpreted using SHAP, PDPs, ICE plots, and Taylor diagrams, providing clear insights into the influence of critical mix parameters. The research has the following conclusions:

- PSO-optimized XGB provided the most accurate predictions, achieving high performance with an R^2^ ≈ 0.82 for Marshall Stability and R^2^ ≈ 0.83 for Marshall Flow.
- SHAP analysis quantitatively identified bitumen content and plastic content as the most influential features affecting MS and MF predictions.
- PDP results indicated optimal plastic particle sizes (2.5–4 mm) and bitumen content (around 5.3–5.5%) to maximize stability and achieve balanced flow properties.
- ICE analysis revealed detailed, instance-specific insights: stability improved by approximately 10–15 kN at optimal plastic sizes (2.5–4 mm), increased by about 8–10 kN beyond 20% plastic content, and showed a stability gain of up to 8–12 kN within optimal aggregate sizes (14–18 mm). Flow values increased notably (3–4 mm) above 20% plastic content and showed incremental increases (1.5–2 mm) between 4 and 6.5% bitumen content, highlighting specific thresholds and sensitivities.
- Taylor diagram analysis validated the superior generalization capability of the PSO-optimized XGB model, showing minimal error and high correlation (> 0.92) with observed values.

Overall, this study demonstrates the potential of optimized and interpretable machine learning models to guide the design of durable, sustainable, and cost-efficient asphalt pavements. By identifying critical thresholds for plastic particle size, plastic content, and bitumen content, the results support informed decision-making in practical design, reducing trial-and-error experimentation and material waste. The findings also carry broader implications for enhanced road safety through pavements with higher stability and controlled flexibility, environmental sustainability via the beneficial reuse of waste plastics, and economic efficiency by minimizing maintenance and life-cycle costs. Future research should prioritize expanding the dataset with field performance observations across diverse traffic and climatic conditions, while also incorporating additional performance indicators such as rutting and fatigue resistance and categorical variables like plastic type and mixing method. Multi-site field trials will be particularly important to externally validate and, if necessary, recalibrate the models against in-service pavement performance (e.g., rutting progression, cracking, and ride quality), thereby ensuring their robustness and practical applicability in pavement design and asset management.

### Declaration of generative AI and AI-assisted technologies in the writing process

During the preparation of this work the authors used ChatGPT only to improve readability and language of the work. After using this tool, the authors reviewed and edited the content as needed under strict human oversight and take full responsibility for the content of the publication.

## Data Availability

The data is available from the corresponding author upon request.
